# Diosmin or Hesperidin? Comparison of Antioxidative Action of Two Venoactive Flavonoids in Type 1 Diabetic Rats

**DOI:** 10.3390/ijms262311252

**Published:** 2025-11-21

**Authors:** Weronika Borymska, Sławomir Borymski, Maria Zych, Sławomir Dudek, Ilona Kaczmarczyk-Żebrowska

**Affiliations:** 1 Department of Pharmacognosy and Phytochemistry, Faculty of Pharmaceutical Sciences in Sosnowiec, Medical University of Silesia, Katowice, Jagiellońska 4, 41-200 Sosnowiec, Poland; mzych@sum.edu.pl (M.Z.); sdudek@sum.edu.pl (S.D.); izebrowska@sum.edu.pl (I.K.-Ż.); 2 Faculty of Natural Sciences, Institute of Biology, Biotechnology and Environmental Protection, University of Silesia in Katowice, Jagiellońska 28, 40-032 Katowice, Poland; slawomir.borymski@us.edu.pl

**Keywords:** diosmin, hesperidin, oxidative stress, type 1 diabetes, rats, venoactive drugs, comparative analysis, flavonoids

## Abstract

Diabetes mellitus and chronic venous disease (CVD) are multifactorial, long-lasting diseases. Although usually considered separately, they often coexist, and individuals with diabetes are more prone to CVD development. Despite different etiology, CVD and diabetic vascular complications share several pathomechanisms, and oxidative stress is one of them. In this study the antioxidative potential of two venoactive flavonoids—diosmin and hesperidin—in the course of type 1 diabetes was compared for the first time. Type 1 diabetic rats were treated with diosmin or hesperidin, each at two doses, 50 and 100 mg/kg, for four weeks. In order to evaluate the antioxidative potential of tested compounds, the antioxidative enzyme activity (superoxide dismutase, catalase and glutathione peroxidase), thiols homeostasis, oxidative status markers (total antioxidative response—TAR, total oxidative status and oxidative stress index—OSI), and oxidative damage markers (advanced oxidation protein products and malondialdehyde) in the serum were measured. Diabetes caused disturbance in the serological redox homeostasis, especially by decreasing enzyme activity and TAR while increasing levels of oxidative damage markers and OSI, increasing advanced glycation end products (AGEs) levels, as well as altering carbohydrate and lipid metabolism. Flavonoids improved the majority of lipid metabolism markers and reduced AGEs with no effect on glycemia. In the context of oxidative stress, their effect was moderate and dose-dependent, and better potency of hesperidin over diosmin was noted, both in individual and multivariate analyses of the parameters. The collective analysis of all parameters led to the conclusion that both diosmin and hesperidin can be considered complementary agents averting negative impact of diabetes due to their multi-faceted actions, including antioxidative properties.

## 1. Introduction

Diabetes mellitus (DM) is a multifactorial, long-lasting metabolic disease characterized by increased glucose levels. It affects more and more people worldwide causing increased healthcare costs [1,2,3]. This disease poses a risk of the development of severe complications, which can be divided into two main types: macrovascular (occurring in large blood vessels), such as cerebrovascular, ischemic heart, or peripheral vascular diseases, and microvascular (affecting small blood vessels), including various neuropathies, retinopathies, nephropathies, or gastroparesis. In addition, there are complications which are combinations of these two above mentioned types, e.g., diabetic foot and erectile dysfunctions [1,4]. Even though DM therapy and management are usually sufficient, the occurrence of complications remains high [5], and these complications are responsible for diabetes-related morbidity and mortality [4].

Chronic venous disease (CVD), like diabetes, is also a disease with many factors involved in its development, and it also generates high healthcare costs. This progressive and persistent disorder in which blood return is compromised affects veins and manifests itself by a broad spectrum of clinical symptoms—from varicose veins to venous ulcerations [6]. Despite the fact that these two diseases are commonly considered two separate entities, DM and CVD frequently appear together, and, what is more, diabetes occurs twice as often in patients with CVD [5].

One of the factors mentioned in CVD development is oxidative stress, which affects the endothelium, leading to its dysfunction [5,6,7]. Hyperglycemia is known to be an important reason of reactive oxygen species (ROS) overproduction, causing oxidative stress and, as a result, diabetic complications, also in the vascular endothelium [8,9]. Even though the etiology of CVD and diabetic vascular disorders is different, in the pathophysiology of both, especially of CVD and microvascular diabetic complications, similar mechanisms are mentioned [5,10]. Moreover, as mentioned above, these two morbidities often coexist; therefore, joint treatment could benefit patients suffering from DM and its complications and/or CVD [5].

There are several therapeutic strategies for CVD treatment, and one of them is the use of so-called venoactive (or phlebotropic, phlebotonic) drugs [11,12]. Among these drugs are plant-derived flavonoids, such as diosmin and hesperidin. These two flavonoids, used separately or in combination, have proven effectiveness in CVD treatment [11,13,14,15,16], since they improve venous tone and reduce venous-related symptoms [11].

**Figure 1:**
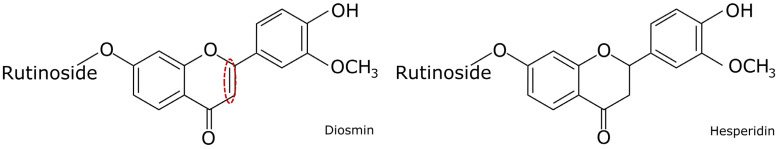
Structure of diosmin (left) and hesperidin (right). The difference between compounds is marked red.

Diosmin and hesperidin are two naturally occurring flavonoids mainly found in citrus fruits. Both are glycosides, precisely 7-O-rutinosides, of diosmetin and hesperetin, respectively. They differ from each other by the presence of a double bond in the central ring (Figure 1) [17]. These two flavonoids reveal numerous pharmacological activities, but they are especially known for their venoactive properties. Since they are considered non-toxic substances, they are popular as medications and dietary supplements [16]. Physico-chemical studies revealed that these two compounds show similar solubility in hydrophilic dissolvents (they are rather insoluble) [18] and have comparable drug-like properties and pharmacokinetic parameters connected with absorption [19]. Nonetheless, the pharmacokinetic studies conducted in humans exposed that diosmetin (an aglycon which is absorbed after diosmin oral administration) has longer half-life elimination [20] than hesperetin—an aglycon of hesperidin [21]. Also, the structure–activity relationship analyses conducted on numerous flavonoids showed that unsaturation of the central ring, together with an oxo functional group, are important features determining the antioxidative properties of the compound [22], suggesting diosmin is a better antioxidant than hesperidin.

These two agents have well-described protective effects against DM and diabetic-related complications in animal models and also reveal a beneficial impact on diabetic patients. The results of these research can be found in numerous original works, but also are presented in review articles [23,24,25,26,27,28,29]. This activity stems, among others, from their antioxidative properties [24,30], but can also result from modulation of crucial metabolic pathways related to diabetes [19].

There are two main types of diabetes: type 1 and type 2. Type 2 diabetes is more common, but type 1 predominantly manifests early in the childhood, and therefore its duration is longer, and such patients are more prone to developing vascular complications earlier [1]. The main strategy for type 1 diabetes treatment is insulin therapy, since in this type of diabetes there is an autoimmune destruction of pancreatic β cells and lack of insulin secretion. Nevertheless, sometimes this therapy is not enough for patients with type 1 diabetes, as insulin resistance can also be observed in these patients, and this phenomenon is responsible for insufficient glycemic control and hence the development of complications, including angiopathies. For this reason, and to improve the general outcome of the therapy, non-insulin drugs are implemented as adjunct agents in patients with type 1 diabetes [31]. In addition, insulin therapy, despite the fact that new analogs are developed, does not completely reflect the physiological action of endogenous insulin and still holds the risk of hypoglycemia episodes [32]. Non-insulin antidiabetic drugs also possess several side effects typical for each class of drugs, and sometimes it is hard to achieve therapeutical goals using single-drug therapy [33]. Currently, more and more studies highlight the beneficial role of antioxidant supplementation in addition to conventional diabetes therapy, indicating its important role in complications management [34]. A cohort study in adults with diabetes indicated that higher dietary antioxidant intake was associated with lower mortality risk, regardless of the cause of death [35]. The venoactive flavonoids diosmin and hesperidin, being dietary antioxidants, also were proven to benefit subjects with type 1 diabetes, including laboratory animals, in which they improved numerous parameters [36,37,38,39,40,41], and patients, in whom administration of a diosmin and hesperidin combination resulted in a slight improvement in glycation, with no side effects observed [42].

The majority of published research regarding the effect of diosmin or hesperidin on diabetic complications or CVD concerns only one of these flavonoids given to experimental animals or patients (only one of them was administered and the second was not used in the study), or the intervention encompassed a combination of these two substances together. Data in which these two compounds are compared in the treatment of DM or CVD or any other condition is scarce. There are reports comparing the effect of diosmin and hesperidin on neuropathic pain [43] and chemically-induced carcinogenesis of the bladder [44], colon [45], esophagus [46], or oral cavity [47], and their antioxidative effect in vivo in several organs of rats with an acrylamide-induced oxidative damage [48], in vitro together with their antiglycating properties [49], ex vivo on the sex-specific relaxation of the human umbilical vein [50], as well as in clinical conditions, on neuropathy and metabolic profile in patients with diabetes with metabolic syndrome [51]. These two compounds were also compared in terms of being ligands for phosphatidylinositol 3-kinase (PI3K) in in silico studies. Moreover, in the same research, at the in vivo stage, type 2 diabetic rats were treated with diosmin or hesperidin and polyflavonoids extracted from lemon peel extract. The results encompassing serological parameters connected with carbohydrates and lipids homeostasis, oxidative damage, and inflammation in the liver and pancreas, as well as molecular analyses regarding the PI3K/Akt/FOXO1 pathway in these organs obtained for diosmin or hesperidin-treated animals, were used for comparison with the lemon peel extract polyflavonoids group [19].

In our study we focused on comparative analysis of these two flavonoid glycosides in the context of oxidative stress in type 1 diabetes conditions. As mentioned above, diabetes and chronic venous disease are two conditions that often coexist [5]; hence, it is highly probable that patients with diabetes suffering from CVD could be advised to take venoactive medications, such as diosmin and/or hesperidin, in order to relieve venous symptoms. To the best of our knowledge, there are no published works, except for our conference paper [52], comparing the antioxidative properties of these two venoactive drugs in type 1 diabetic conditions; therefore, the aim of this study was to investigate and compare the effect of diosmin and hesperidin against oxidative stress in rats with experimentally-induced type 1 diabetes.

## 2. Results

### 2.1. Effect of Tested Flavonoids on Carbohydrate Metabolism and AGEs Levels

**Figure 2:**
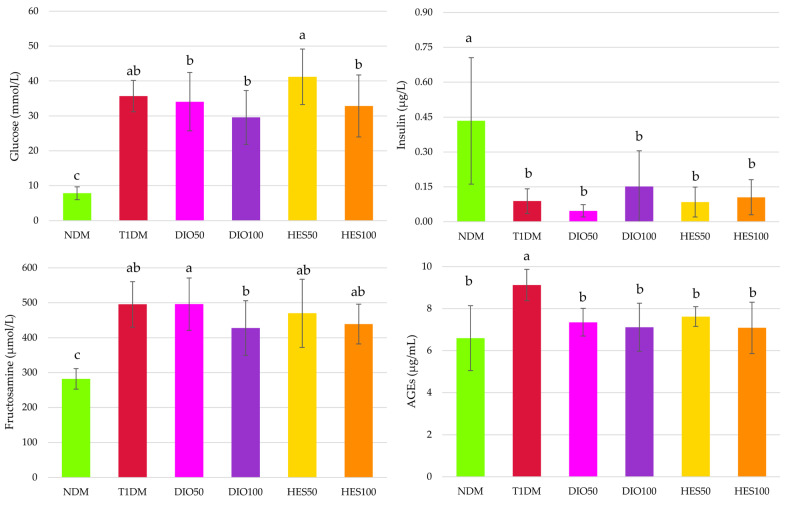
Effect of diosmin and hesperidin on carbohydrate metabolism markers and advanced glycation end products in type 1 diabetic rats. NDM—control rats in which diabetes was not induced (n = 9), T1DM—control rats in which type 1 diabetes was induced (n = 8), DIO50—type 1 diabetic rats administered with diosmin at a dose of 50 mg/kg (n = 9), DIO100—type 1 diabetic rats administered with diosmin at a dose of 100 mg/kg (n = 9), HES50—type 1 diabetic rats administered with hesperidin at a dose of 50 mg/kg (n = 8), HES100—type 1 diabetic rats administered with hesperidin at a dose of 100 mg/kg (n = 9), AGEs—advanced glycation end products. Results in the graphs are presented as arithmetical means ± standard deviation. Statistical significances were evaluated with one-way ANOVA followed by Fisher’s LSD post hoc test. The letters in the superscripts indicate statistical significances. Values presented in individual panels sharing at least one letter reveal no statistically significant differences at *p* < 0.05.

Diabetes induction resulted in severe changes in the carbohydrate metabolism in all streptozotocin-treated animals. Glucose and fructosamine levels were significantly elevated when compared to healthy, non-diabetic rats, while insulin levels decreased. None of the flavonoids, despite the dose, affected these parameters when compared with diabetic controls (T1DM). Nevertheless, when glucose levels in the serum were considered, it was observed that the administration of hesperidin at the dose of 100 mg/kg resulted in a significant reduction of this parameter in comparison with the group receiving the lower dose of this flavonoid—50 mg/kg. Also, both the doses of diosmin, administered to the diabetic rats, showed significantly lower glucose levels in the serum than the rats treated with hesperidin at the dose of 50 mg/kg. Moreover, fructosamine levels in the diosmin-treated groups, even though not different from the levels recorded in the T1DM rats, showed a dose-dependent manner of reduction—in the DIO100 rats, fructosamine levels were significantly lower than in the DIO50 rats. Advanced glycation end products (AGEs) levels in the serum of the diabetic animals (T1DM group) also increased in comparison with those in nondiabetic rats (NDM group), but, contrary to glucose and fructosamine levels, administration of both diosmin and hesperidin at both the doses (i.e., 50 and 100 mg/kg) for 28 days reduced the AGEs levels in the serum of the diabetic animals (Figure 2).

**Figure 3:**
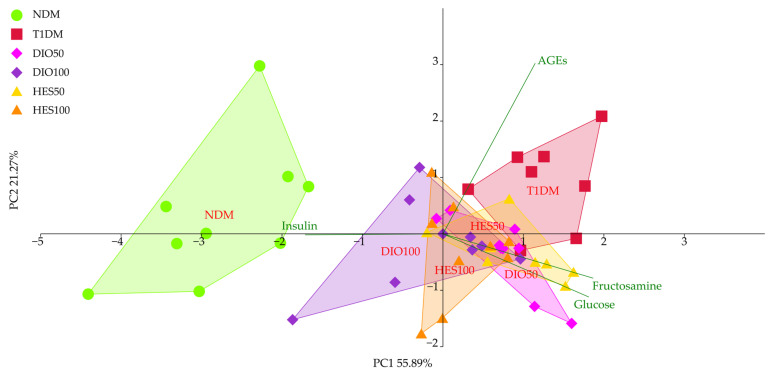
PCA for the glycemic markers from the serum. NDM—control rats in which diabetes was not induced (n = 9), T1DM—control rats in which type 1 diabetes was induced (n = 8), DIO50—type 1 diabetic rats administered with diosmin at a dose of 50 mg/kg (n = 9), DIO100—type 1 diabetic rats administered with diosmin at a dose of 100 mg/kg (n = 9), HES50—type 1 diabetic rats administered with hesperidin at a dose of 50 mg/kg (n = 8), HES100—type 1 diabetic rats administered with hesperidin at a dose of 100 mg/kg (n = 9).

The PCA for the glycemic parameters showed a significant discrimination of the experimental groups across axis PC1, explaining almost 56% of the total observable variation (MANOVA, *p* < 0.001) (Figure 3, Table 1). The NDM group separated to the left of the PC1, while the T1DM group clustered on the opposite side, to the right. The DIO100 and HES100 groups showed significant separation from both the T1DM and NDM groups, clustering in the middle of the plot. The DIO50 and HES50 groups did not show significant separation from the T1DM group; hence, no improvement was observed for these groups when it comes to parameters associated with glycemia. Only the DIO100 group showed statistically significant separation from the T1DM group when it comes to PC2; however, this axis corresponded to just over 21% of the observable variation. The major sources of variation for PC1 were insulin, fructosamine, and glucose, whereas the separation across PC2 was mostly affected by AGEs.

### 2.2. Effect of Tested Flavonoids on Lipid Metabolism and Hepatic Functions

**Figure 4:**
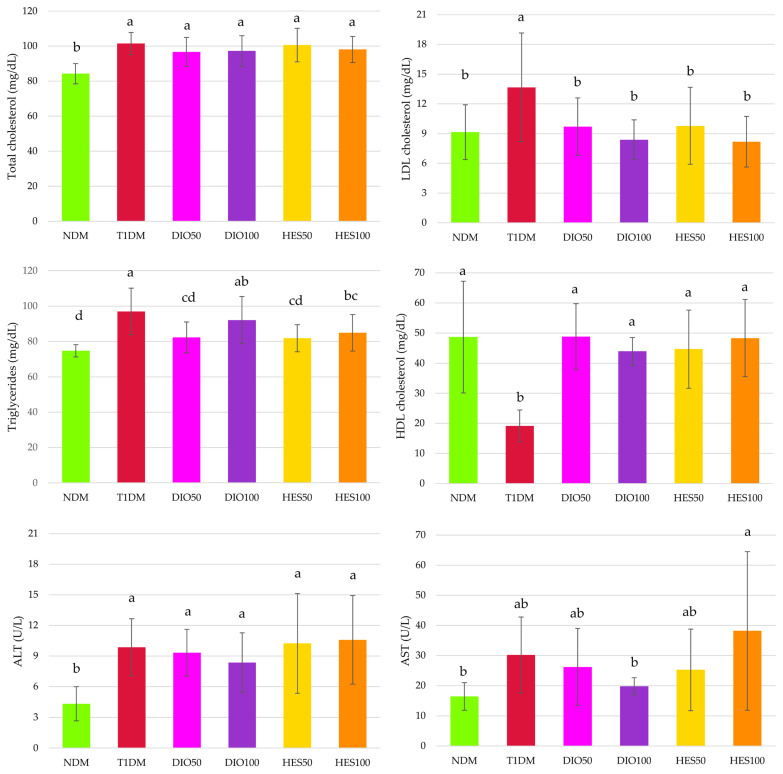
Effect of diosmin and hesperidin on lipid metabolism and hepatic function markers and aminotransferases activity in type 1 diabetic rats. NDM—control rats in which diabetes was not induced (n = 9), T1DM—control rats in which type 1 diabetes was induced (n = 8), DIO50—type 1 diabetic rats administered with diosmin at a dose of 50 mg/kg (n = 9), DIO100—type 1 diabetic rats administered with diosmin at a dose of 100 mg/kg (n = 9), HES50—type 1 diabetic rats administered with hesperidin at a dose of 50 mg/kg (n = 8), HES100—type 1 diabetic rats administered with hesperidin at a dose of 100 mg/kg (n = 9). Results in the graphs are presented as arithmetical means ± standard deviation. Statistical significances were evaluated with one-way ANOVA followed by Fisher’s LSD post hoc test. The letters in the superscripts indicate statistical significances. Values presented in individual panels sharing at least one letter reveal no statistically significant differences at *p* < 0.05.

A negative effect of diabetes induction on lipid profile in rats was noted. Induction of type 1 diabetes resulted in an increase in total cholesterol, LDL cholesterol, and triglyceride levels as well as in a decrease in HDL cholesterol levels. Administration of diosmin and hesperidin did not affect the level of total cholesterol, but both these flavonoids reduced the level of LDL cholesterol and elevated HDL cholesterol levels. Also, triglyceride levels were positively affected by these flavonoids; hesperidin at both the doses and diosmin at the lower 50 mg/kg dose reduced this parameter when compared to the T1DM rats. As far as transaminases (alanine and aspartate—ALT and AST) are concerned, ALT activity was significantly higher in all diabetic rats as compared to the NDM rats. The flavonoid administration did not reduce it, while AST activity was not elevated except in the HES100 group, in which its activity was significantly higher than in the NDM or DIO100 rats, but did not differ from the activity reported in the T1DM rats (Figure 4).

**Figure 5:**
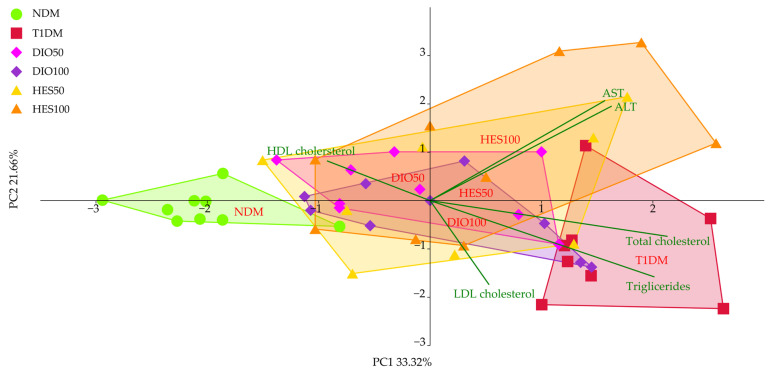
PCA for the lipid metabolism markers and hepatic functions from the serum. NDM—control rats in which diabetes was not induced (n = 9), T1DM—control rats in which type 1 diabetes was induced (n = 8), DIO50—type 1 diabetic rats administered with diosmin at a dose of 50 mg/kg (n = 9), DIO100—type 1 diabetic rats administered with diosmin at a dose of 100 mg/kg (n = 9), HES50—type 1 diabetic rats administered with hesperidin at a dose of 50 mg/kg (n = 8), HES100—type 1 diabetic rats administered with hesperidin at a dose of 100 mg/kg (n = 9).

The distribution of the experimental groups resulting from lipid metabolism and hepatic functions markers revealed a clear separation between the untreated control (NDM) and diabetic control (T1DM) groups, which separated to the left and to the right across the PC1 axis, respectively (Figure 5, Table 2). The remaining groups (DIO50, DIO100, HES50 and HES100) grouped in the center of the plot, which suggests the improvement in lipid markers upon application of diosmin and hesperidin. All of these effects were statistically significant across PC1, explaining over 33% of observable variation (MANOVA, *p* < 0.001). As for the PC2, the effects were more nuanced, with T1DM and NDM clearly separated; however, only the DIO50, HES50, and HES100 groups separated from the diabetic group, indicating a positive change. The PC1 was correlated mostly with total cholesterol, HDL cholesterol, and triglycerides. Roughly equal correlation for PC1 and PC2 was observed for AST and ALT, whereas LDL cholesterol was mostly correlated with the PC2. Thus, the clustering along PC1 and PC2 was affected by the parameters correlated with each of the axes.

### 2.3. Effect of Tested Flavonoids on Oxidative Stress Markers

The activity of three antioxidative enzymes was measured: superoxide dismutase (SOD), catalase (CAT), and glutathione peroxidase (GPx). In diabetic conditions, the activity of SOD and GPx was significantly reduced and administration of diosmin or hesperidin, despite the dose, did not result in elevation of their activity. CAT activity in the T1DM rats was not decreased when compared to the NDM rats. Also, its activity in the DIO50 and DIO100 rats was not different from the CAT activity reported in the NDM or T1DM rats. However, in the HES50 and HES100 rats, CAT activity was significantly higher than in the T1DM and both diosmin-treated groups, and in the HES50 rats, the recorded value was even higher than in the NDM rats (Table 3).

The measurement of thiols content revealed that total thiol content did not differ between all the tested groups, but the level of native thiols, representing free -SH groups, increased significantly in all diabetic groups of rats, except in the HES50 group. Disulfides level in the T1DM rats did not change when compared to the NDM rats; however, their level was significantly lower in the DIO100 and HES100 groups when compared to the NDM rats. No changes in the disulfides level were noted after diosmin or hesperidin administration when their level was compared with T1DM rats. As a result, the native thiols/disulfides ratio calculated from the abovementioned parameters differed only from the values recorded in the NDM rats, in the rats from the DIO100 and HES100 groups, but no differences in this ratio were recorded between the T1DM rats and any of the flavonoid-treated rats (Table 3).

**Figure 6:**
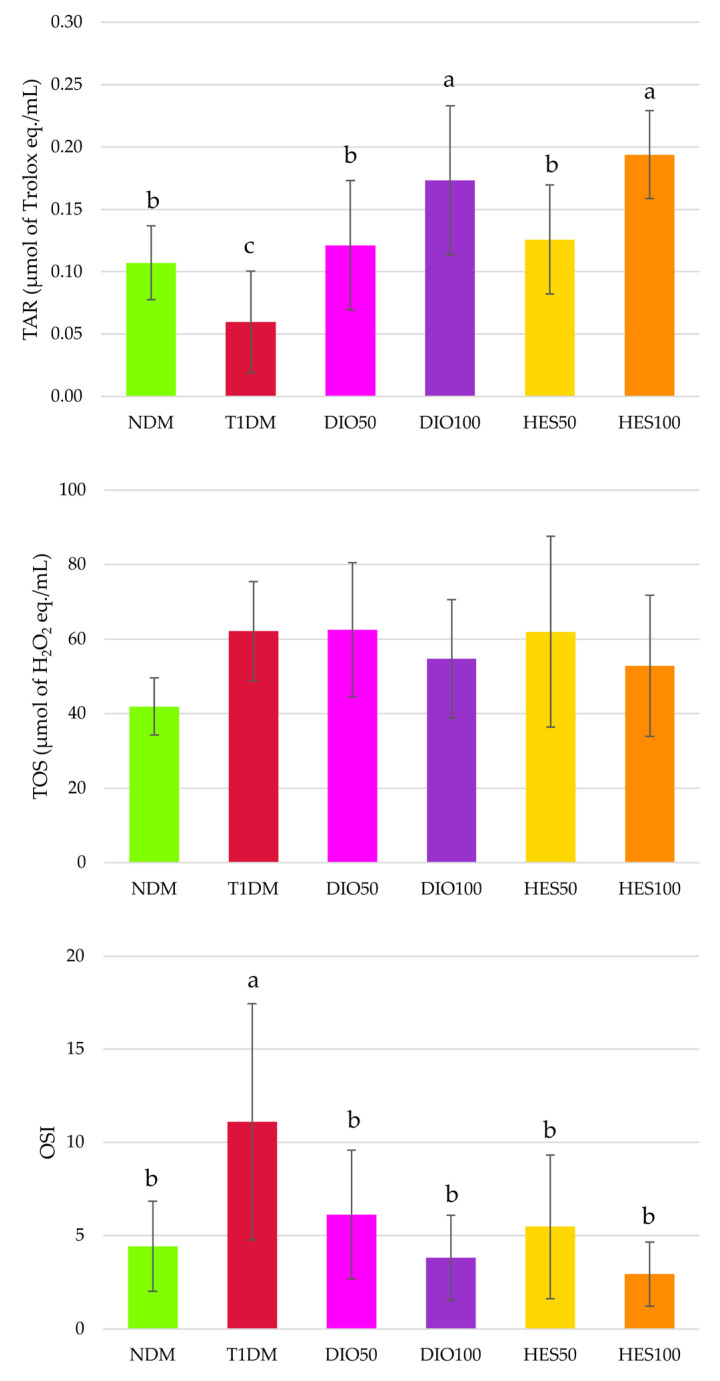
Effect of diosmin and hesperidin on total oxidative stress-related markers in the serum of type 1 diabetic rats. NDM—control rats in which diabetes was not induced (n = 9), T1DM—control rats in which type 1 diabetes was induced (n = 8), DIO50—type 1 diabetic rats administered with diosmin at a dose of 50 mg/kg (n = 9), DIO100—type 1 diabetic rats administered with diosmin at a dose of 100 mg/kg (n = 9), HES50—type 1 diabetic rats administered with hesperidin at a dose of 50 mg/kg (n = 8), HES100—type 1 diabetic rats administered with hesperidin at a dose of 100 mg/kg (n = 9), TAR—total antioxidative response, TOS—total oxidative status, OSI—oxidative stress index. Results in the graphs are presented as arithmetical means ± standard deviation. Statistical significances were evaluated with one-way ANOVA followed by Fisher’s LSD post hoc test. The letters in the superscripts indicate statistical significances. Values presented in individual panels sharing at least one letter reveal no statistically significant differences at *p* < 0.05.

Total antioxidative response (TAR) was significantly lower in the T1DM rats than in the NDM rats, and administration of both diosmin and hesperidin resulted in TAR elevation; moreover, this increase was dose-dependent, since the higher doses (100 mg/kg) revealed higher TAR values than recorded in the NDM, T1DM, DIO50 and HES50 groups. Total oxidative status (TOS) did not change in a statistically significant manner, but the oxidative stress index (OSI) increased after diabetes induction, and administration of both the flavonoids at both the doses restored its values to the level recorded in the NDM rats (Figure 6).

**Figure 7:**
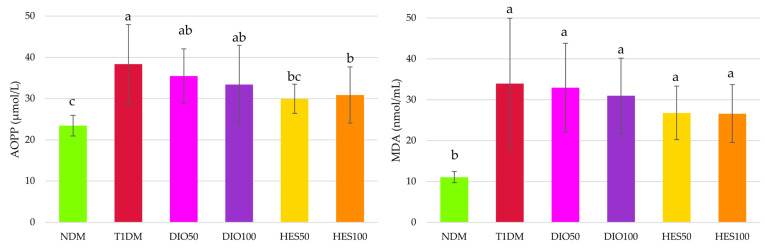
Effect of diosmin and hesperidin on oxidative damage markers in the serum type 1 diabetic rats. NDM—control rats in which diabetes was not induced (n = 9), T1DM—control rats in which type 1 diabetes was induced (n = 8), DIO50—type 1 diabetic rats administered with diosmin at a dose of 50 mg/kg (n = 9), DIO100—type 1 diabetic rats administered with diosmin at a dose of 100 mg/kg (n = 9), HES50—type 1 diabetic rats administered with hesperidin at a dose of 50 mg/kg (n = 8), HES100—type 1 diabetic rats administered with hesperidin at a dose of 100 mg/kg (n = 9), AOPP—advanced oxidation protein products, MDA—malondialdehyde. Results in the graphs are presented as arithmetical means ± standard deviation. Statistical significances were evaluated with one-way ANOVA followed by Fisher’s LSD post hoc test. The letters in the superscripts indicate statistical significances. Values presented in individual panels sharing at least one letter reveal no statistically significant differences at *p* < 0.05.

Diabetes induction in experimental animals resulted in oxidative damage to proteins and lipids. It was noted that advanced oxidation protein products (AOPP) level, representing protein oxidation, significantly increased in the T1DM group, and only hesperidin administration counteracted these changes; however, AOPP levels in hesperidin-treated rats were not significantly lower than in the diosmin-treated animals. The malondialdehyde (MDA) level was elevated in all streptozotocin-treated rats, and none of the interventions affected it (Figure 7).

**Figure 8:**
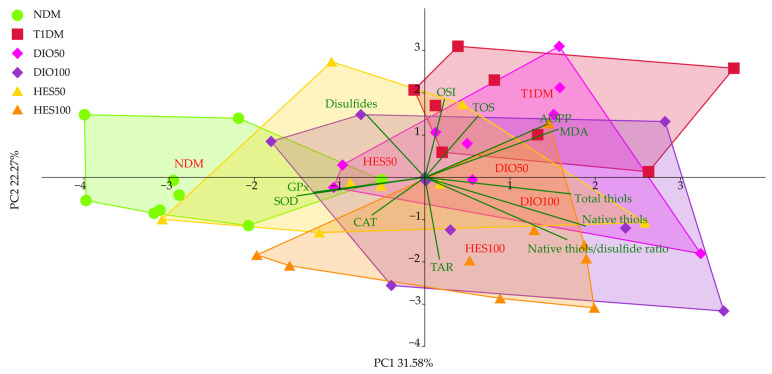
PCA for the oxidative stress-related markers from the serum. NDM—control rats in which diabetes was not induced (n = 9), T1DM—control rats in which type 1 diabetes was induced (n = 8), DIO50—type 1 diabetic rats administered with diosmin at a dose of 50 mg/kg (n = 9), DIO100—type 1 diabetic rats administered with diosmin at a dose of 100 mg/kg (n = 9), HES50—type 1 diabetic rats administered with hesperidin at a dose of 50 mg/kg (n = 8), HES100—type 1 diabetic rats administered with hesperidin at a dose of 100 mg/kg (n = 9).

As for the oxidative state markers from the serum, the PCA showed separation of the NDM group to the left of the plot, away from the T1DM and the rest of the experimental groups across the PC1, explaining over 30% of the total variation observed (Figure 8, Table 4). That said, only the HES50 group proved to be significantly different from the T1DM group across the PC1 (MANOVA, *p* < 0.001), but the direction of the change was not uniform, with the pattern revealed for untreated control (NDM). However, a pattern similar to untreated control was observed for the HES50 group across PC2, explaining over 22% of the total variation. The remaining groups, the DIO50 clustered between NDM and T1DM, hinting at signs of improvement in the oxidative state (MANOVA, *p* < 0.001); however the HES100 and DIO100 groups showed a distinct dissimilarity with the control groups (NDM and T1DM). Total-SH, native-SH, native thiols/sulfide ratio, AOPP, MDA, GPx, CAT, and SOD were associated more with the variation observed across the PC1, whereas OSI, TOS, TAR, and disulfides correlated more with the PC2.

**Figure 9:**
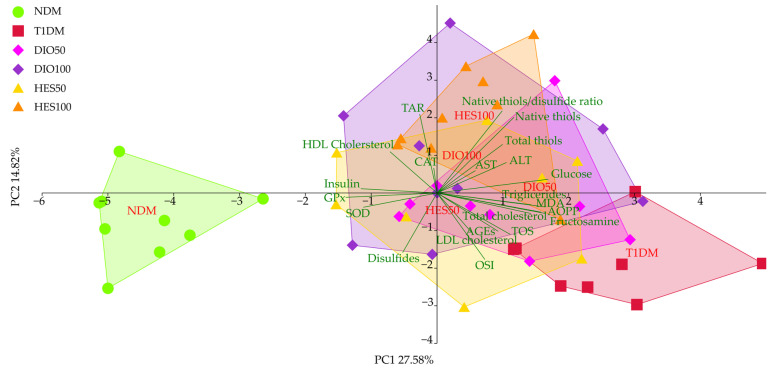
PCA for all tested markers from the serum. NDM—control rats in which diabetes was not induced (n = 9), T1DM—control rats in which type 1 diabetes was induced (n = 8), DIO50—type 1 diabetic rats administered with diosmin at a dose of 50 mg/kg (n = 9), DIO100—type 1 diabetic rats administered with diosmin at a dose of 100 mg/kg (n = 9), HES50—type 1 diabetic rats administered with hesperidin at a dose of 50 mg/kg (n = 8), HES100—type 1 diabetic rats administered with hesperidin at a dose of 100 mg/kg (n = 9).

The PCA for all of the parameters tested from the serum showed separation mainly along axis PC1, responsible for more than 27% of observable variation (Figure 9, Table 5). This separation was statistically significant (*p* < 0.001) for the NDM and T1DM groups, which separated into clusters to the left and to the right of the PC1, respectively (LSD post hoc). The remaining experimental groups, except DIO50, clustered in the middle, between the NDM and T1DM groups. The DIO50 group did not reveal significant separation compared to the T1DM group. The effects associated with PC2 were also statistically significant, with T1DM separating to the lower part of PC2, whereas HES100 clustered in the upper part of the PCA plot, and DIO50 and DIO100 falling in between. T1DM, NDM, and HES50 did not reveal significant differences in separation along PC2. The major sources of variations associated with PC1 were identified as glucose, MDA, AOPP, and fructosamine, as well as GPx, insulin, and SOD. The PC2 separation was mostly affected by the native thiols–disulfide ratio, native thiols and TAR, as well as OSI and disulfides.

## 3. Discussion

In this study we aimed to compare two venoactive drugs, diosmin and hesperidin, in terms of their antioxidative action in the serum of type 1 diabetic rats. Although both these compounds reveal similar features related to their physico-chemical properties and partially pharmacokinetic features, the structure–activity relationship and half-life in the serum indicate that diosmin could be more potent [18,19,20,21,22]. The easiest and least invasive way to monitor patients’ well-being and health condition is to analyze their blood, as this biological fluid circulates through the whole body and can be easily obtained by vein punctuation. More precisely, two fractions of blood are commonly used—plasma and declotted serum [53]. However, in the context of metabolomic studies, serum is recommended over plasma [54]. Type 2 diabetes is more frequent in the population, and numerous animal studies are based on this type of diabetes. Yet, type 1 usually has an earlier onset and complications, including vascular ones, and can affect the patient sooner; hence, we focused on type 1 diabetes, comparing for the first time the effect of these two flavonoids on oxidative stress-related parameters in the rat serum. For this purpose we used high dose of streptozotocin (60 mg/kg) to induce type 1 diabetes, and this model is commonly used in laboratory protocols, as it mimics type 1 diabetes symptoms in humans [55,56].

As we reported before, the diabetic rats in this experiment (control, untreated diabetic rats, and diabetic rats undergoing flavonoid intervention) developed features typical for type 1 diabetes: polydipsia, polyphagia, and polyuria, and their body mass was greatly reduced [57,58]. Administration of diosmin at a higher dose slightly counteracted body mass loss [58].

In our study, severe hyperglycemia and hypoinsulinemia were recorded in all streptozotocin-treated groups of rats. The administered flavonoids did not counteract these pathological changes, but in case of hesperidin, a dose-dependent manner in reducing serum glucose levels was observed. Even though these changes were not enough to be statistically significant in relation to the T1DM rats, the higher dose of hesperidin, 100 mg/kg, seemed to be more potent than the lower, 50 mg/kg dose. Similar observations, that hesperidin acts on glucose levels in diabetic rats in a dose-dependent manner were previously described, but in that study, the glucose level reduction was significant [59]. Diosmin also has been reported to lower blood glucose levels in such a manner [60], but we did not observe any changes in this parameter after diosmin administration, regardless of the dose. Nevertheless, fructosamine levels, a marker representing long-term glycemia status [61], changed in a dose-dependent manner after diosmin treatment—administration of the higher dose, 100 mg/kg, resulted in significantly lower fructosamine levels than after the lower, 50 mg/kg dose. Still these changes were not significant in comparison with the T1DM rats. In a meta-analysis of hesperidin’s effect on blood glucose and insulin levels in human trials, it was noticed that, despite the numerous articles presenting results from animal studies, this flavonoid had no effect on glycemia-related parameters regardless of the health status of the participants (diabetic or not) [62]. To the best of our knowledge, there are no similar meta-analyses on diosmin’s effect in humans. There is, however, one randomized clinical trial in which diabetic patients with metabolic syndrome were enrolled, where the effect of diosmin and hesperidin alone or in combination was assessed on metabolic profile, and fasting blood glucose levels were determined before and after the intervention [51]. It was recorded that both tested flavonoids alone and in combination, contrary to our study, reduced fasting blood glucose levels. However, it should be highlighted that in our experiment, the blood glucose was tested without fasting (random glycemia), and the animals were type 1 diabetic with severe hyperglycemia and hypoinsulinemia, while in the cited trial, patients were type 2 diabetics. Moreover, significant reduction of fasting blood glucose was also noticed in the placebo group of that trial [51]. It is clearly visible that if glycemia-related parameters (glucose level, insulin level, and fructosamine level) are considered, tested flavonoids could not counteract pathological changes in diabetic rats resulting from streptozotocin action.

Hyperglycemia is a direct cause of advanced glycation end products (AGEs) formation. AGEs are products of the non-enzymatic reaction of reducing sugars (such as glucose) with lipids or amino acids on proteins, and are toxic compounds responsible for numerous diseases, including diabetes, and their complications [63,64,65]. In the presented study, AGEs levels in the serum in control diabetic rats were significantly higher than in the nondiabetic controls, and this result is consistent with previous research [66,67]. Administration of diosmin and hesperidin at both the doses efficiently reduced AGEs levels. Diosmin has been reported to reduce serological AGEs levels in vivo in rats with metabolic syndrome [68] and ex vivo in the lenses exposed to high-glucose environment [69]. It has been also proven that hesperidin may inhibit AGEs formation in vitro [70] and in vivo in diabetic conditions—in the serum of pregnant rats [71] or retina of type 1 diabetic rats [72]. Both these flavonoids significantly reduced AGEs levels in the serum of fructose + streptozotocin-induced type 2 diabetic rats [19]. In an in vitro study comparing several venoprotective flavonoids, it was shown that both diosmin and hesperidin effectively inhibited AGEs formation, and in the case of hesperidin, its effect was even greater than of the reference substance—aminoguanidine—while diosmin revealed a comparable effect to this compound [49]. This feature of both diosmin and hesperidin is highly important in the context of diabetes and its complications, as well as of CVD. It is well documented that AGEs resulting from hyperglycemia may accumulate in the body causing complications, including microvascular complications [73,74], since increased AGEs are related to endothelial dysfunction [75]. Therefore, AGEs are potential molecular targets for inhibition of diabetes-related vascular problems [76]. Even though the tested flavonoids did not affect individual carbohydrate metabolism markers in type 1 diabetic rats, when these parameters and advanced glycation end products (AGEs) are considered together in principal component analysis, it can be seen that the higher diosmin and hesperidin doses affect them in a statistically significant manner, suggesting that these flavonoids can be beneficial in diabetic conditions, also in the context of glycemia. This beneficial aggregated effect of diosmin and hesperidin on glycemia could be explained by the modulatory effect of both these flavonoids on the phosphatidylinositol 3-kinase/protein kinase B/forkhead box-O1 (PI3K/Akt/FOXO1) pathway. Both substances were proven to be ligands for PI3K in in silico molecular docking studies and in vivo, where they enhanced gene expression for PI3K and FOXO1 in the liver, as well as PI3K, p-AKT, FOXO1, and adenosine monophosphate-activated protein kinase (AMPK) in the pancreas of type 2 diabetic rats. Moreover, in the cited study, diosmin and hesperidin increased gene expression for the glucose transporters GLUT2 and GLUT4 in both these organs in diabetic animals. Altogether, their effect in fructose and streptozotocin-induced type 2 diabetic rats was assumed beneficial when administered separately, and even greater when the treatment involved polyflavonoids (containing diosmin, hesperidin, biochanin A, hesperetin, and quercetin) from lemon peel extract, suggesting their synergistic effect on the PI3K/Akt/FOXO1 signaling route [19].

Diabetes affects not only carbohydrate metabolism, but also strongly influences lipid metabolism, causing disruptions in cholesterol, HDL, LDL, and triglycerides levels—so called diabetic dyslipidemia [77,78]. It also disturbs hepatic functions, also in type 1 diabetic patients, especially with poor glycemia control, causing hepatopathies, such as glycogenic hepatopathy, in which altered activities of AST or ALT are hallmarks [79]. In our experiment, lipid metabolism and ALT activity were negatively changed by diabetes induction, which is in line with observations from already published studies [80,81,82]. Diosmin and hesperidin failed to reduce total cholesterol levels and restore the ALT activity, but they successfully reduced LDL cholesterol levels and increased HDL cholesterol levels, and, except for diosmin at a dose 100 mg/kg, reduced triglyceride levels. Diosmin was reported earlier to reduce both cholesterol and triglyceride levels in diabetic rats [36] and regulate lipid profile in rats with metabolic syndrome [68] and experimental diabetes [83]. There is a proposed mechanism for lipid metabolism regulation by diosmin, which was studied in vitro on hepatoblastoma cell line G2 (HepG2) and 3T3-L1 cells. The study confirmed that diosmin reduced lipid content via activation of the AMP-activated protein kinase (AMPK) pathway and, subsequently, phosphorylation of acetyl-CoA carboxylase (ACC), one of the key regulators of cholesterol and fatty acid synthesis [84]. As for hesperidin’s effect on lipid metabolism markers, our results also stay in line with previously published works. There was an improvement in the lipid profile in diabetic rats treated with 100 mg/kg, with no effect on AST or ALT [85] or a significant recovery in these parameters after treatment with hesperidin at a dose of 50 mg/kg in type 2 diabetic rats [86]. The authors of the latter study made an effort to understand the possible mechanisms underlying the antidyslipidemic effect of hesperidin assaying the activity of the hepatic hydroxymethylglutaryl-CoA (HMG-CoA) reductase. HMG-CoA reductase, like ACC, plays an important role in cholesterol synthesis, hence it is a target for drugs (which inhibit its activity) against hypercholesterolemia and dyslipidemia. The study confirmed that hesperidin is an inhibitor of the hepatic HMG-CoA reductase [86]. Both diosmin and hesperidin also improved the lipid profile in fructosamine + streptozotocin-induced type 2 diabetic rats [19]. LDL in diabetic conditions is highly prone to oxidation and glycation. In an in vitro study conducted on human plasma incubated with several flavonoids, it was shown that flavonoids may bind to LDL particles, decreasing their susceptibility to oxidation and glycation in a high-glucose environment [87]. However, neither diosmin nor hesperidin was tested in that study; hence, it can only be hypothesized that in the case of these two flavonoids, a similar mechanism is probable. Both diosmin and hesperidin administered individually or in combination have also been reported to improve triglycerides and LDL levels in type 2 diabetic patients with metabolic syndrome in a randomized controlled clinical trial, with no effect on HDL [51]. Reducing triglycerides levels could also be important in the context of CVD, since it was observed that in patients suffering from chronic venous insufficiency (CVI, a form of CVD with more advanced symptoms), triglycerides content was significantly higher than in patients without CVI, and it was chosen as an independent predictor of CVI development [88]. Interestingly, when all markers related to lipid metabolism were subjected to PCA, the lower doses of both diosmin and hesperidin performed better than the higher doses, showing that in the case of lipids, there is a reversed dose-dependent effect.

Hyperglycemia is the reason for overproduction of reactive oxygen species (ROS) and oxidative stress development. In normoglycemic conditions, glucose is metabolized via several pathways, with glycolysis as a main route, and secondary pathways such as pentose phosphate, glucuronate, and glycogen ones. All these routes may generate ROS, but in physiological concentrations that are not important in the context of oxidative stress. Nevertheless, when glucose levels increase in hyperglycemia, several other pathways are upregulated, such as the polyol pathway, hexosamine pathway, or protein kinase C route, which strongly contribute to ROS overproduction and oxidative stress development. Moreover, glucose can undergo spontaneous reactions and be involved in AGEs formation and, in turn, oxidative stress progression. Numerous studies, both experimental and clinical, highlight the fact that this hyperglycemia-induced oxidative stress is responsible for the development of diabetic complications, including vascular complications and endothelial dysfunctions. Except for ROS overproduction during diabetes, the endogenous antioxidative system can also be compromised—alterations in non-enzymatic antioxidants and impaired activity of antioxidant enzymes often is observed [8,89]. The results in our study show that the oxidative stress index (OSI) was greatly elevated in the serum of the control diabetic rats, indicating severe hyperglycemia-induced oxidative stress in the animals. Also, a significant decrease in SOD and GPx activity was observed, as well as reduced total antioxidative response. No significant change in CAT activity was observed. It should be highlighted that the response of antioxidative enzymes to oxidative stress can differ (their activity may be elevated, depleted, or unchanged) and depends, among other things, on the intensity of the oxidative stress [90]. Alterations in antioxidative enzyme activity and/or total markers representing oxidative stress such as TAR or OSI were previously reported in animal studies [91,92] and in type 1 diabetic patients [93,94]. Also, markers related to oxidation of macromolecules—i.e., proteins and lipids, namely the advanced oxidation protein products (AOPP) and malondialdehyde (MDA)—were significantly elevated in the serum of the diabetic animals. Such alterations were also reported before in diabetic animals and patients [92,93,94,95,96,97]. Administration of diosmin and hesperidin at both the doses did not counteract pathological changes in SOD or GPx—their serological activity remained reduced in comparison to NDM rats and was not elevated above the activity recorded for T1DM rats. Also, the MDA level was not affected upon diosmin or hesperidin treatment. Lack of changes in SOD activity and the MDA levels in rats treated with diosmin or hesperidin could be a result of the fact that neither of these flavonoids affected glycemia (both glucose level and fructosamine level) to such an extent that they counteracted the effect of the diabetes. The study conducted by Astari et al. in patients with type 1 diabetes showed that there is a correlation between glycated hemoglobin (HbA1c) and these two oxidative stress-related markers: Hb1Ac was positively correlated with MDA levels and negatively correlated with SOD activity [98]. Since both fructosamine and HbA1c are markers representing long-term glycemia state and their levels correlate with each other [99], correlation between the SOD, MDA, and fructosamine measured in our study could be similar to that observed for HbA1c. Diosmin also failed to reduce the AOPP level in the serum of diabetic rats, but hesperidin at both the doses effectively decreased this marker when compared to the diabetic, untreated control group. In our previous research we showed that diosmin can reduce its level in the lenses of T1DM rats [58], and in another study diosmin reduced hepatic AOPP levels in rats with fibrosis induced by irradiation [100]. It was also shown that diosmin may protect human serum albumin from oxidative damage by binding to it [101]. Contrary to these findings, we did not observe a beneficial effect of diosmin administration on AOPP in the serum of T1DM rats. To the best of our knowledge, there is no data regarding the effect of diosmin on AOPP levels in the serum of diabetic subjects; thus, we cannot compare our results with the literature. Even though we also cannot directly compare our results for hesperidin action on AOPP level with other research conducted on diabetic models, similar observations on reducing this parameter after hesperidin treatment were noted in the plasma of high-fat-diet-fed dyslipidemic rats, in which glucose metabolism was also negatively affected—100 mg/kg of hesperidin, reduced AOPP level, when compared to untreated rats [102]. In our research, thiol homeostasis was also analyzed. Total thiol content was not affected significantly by diabetes induction or flavonoids administration. Similar observations were made previously in a study in which diabetic animals were treated with imidazolyl thiazolidinedione. The serum levels of total thiols were not affected by streptozotocin injection or drug administration [103]. Interestingly, the level of native thiols, i.e., containing free -SH groups, was significantly higher in the serum of the T1DM rats than in the serum of control, nondiabetic animals, and this level remained high after diosmin or hesperidin interventions. There are many molecules containing free -SH groups, such as cysteine, γ-glutamylcysteine, and coenzyme A, but reduced glutathione (GSH) is the most abundant one [104]. Many experimental studies conducted in diabetic animals reported that native thiols, especially GSH level, were depleted in the serum [105,106,107,108]; therefore, our results contrast with other research conducted in diabetic animals. Nevertheless, the results obtained during our experiment are in line with studies conducted on patients with diabetes—in women with type 2 diabetes, native thiols content in the serum was significantly higher than in individuals with prediabetes or healthy participants [109]; also, patients with diabetes and senile cataract were characterized by an increased GSH level in the serum [95]. GSH can be utilized by GPx in order to form the disulfide form of glutathione, GSSG [110], and in our study the activity of GPx was reduced in the serum of the diabetic rats—untreated and after flavonoid intervention. This lowered GPx activity could be a reason for elevated native thiols content and lack of significant changes in disulfides content. Also, elevated native thiol levels could be some kind of compensatory mechanism to combat oxidative stress, but this theory needs further investigation. Oxidative stress in our study was also depicted by total markers—total antioxidant response (TAR), total oxidative status (TOS), and oxidative stress index (OSI), which was calculated from these two parameters. As mentioned above, TAR was reduced in the serum of the T1DM rats, and OSI was significantly higher, despite the fact that TOS was unchanged. Administration of diosmin and hesperidin resulted in significant OSI reduction, indicating that these flavonoids are capable of reducing oxidative stress. The in vitro study conducted by Bednarska et al. showed that both diosmin and hesperidin are potent antioxidants, but hesperidin exhibited stronger antioxidative properties in both the FRAP and ABTS assays than diosmin. Also, the effect for both these flavonoids was dose-dependent [49]. We also noticed that TAR was dose-dependent after flavonoid treatment—after administration of diosmin or hesperidin at the dose of 100 mg/kg, the TAR value in the serum was significantly higher than after treatment with the dose of 50 mg/kg, and was even higher than in the control, nondiabetic animals. Better performance of hesperidin over diosmin in the context of oxidative stress parameters was visible in individual markers, e.g., in CAT activity, which was significantly higher than in the NDM and T1DM rats, or in AOPP levels. Likewise, the PCA revealed a similar pattern with regard to combined oxidative stress markers, AOPP being the common element between the two analyses. This observation seems to stay in contrast with the structure–activity relationship data, suggesting diosmin is the more potent antioxidant [22]; thus, this phenomenon needs further investigation. Additionally, in PCA, other markers, such as SOD, GPx, MDA, and thiols, were shown to be significant. In PC2, both doses of hesperidin and the higher dose of diosmin resulted in a distinct separation from a cluster formed by T1DM, and the cluster formed by the higher dose of hesperidin was also significantly separated from the NDM cluster along this axis. Dose-dependency for both flavonoids on oxidative stress is also visible. Diosmin and hesperidin were also proven to be good antioxidative agents in the liver and pancreas of type 2 diabetic rats, and, according to the molecular analyses, their modulating effects on the PI3K/Akt/FOXO1 pathway may contribute to their antioxidative and anti-inflammatory effect [19]. The antioxidative action of diosmin and hesperidin is also crucial in the context of venous complications and CVD, since oxidative stress is one of the factors accountable for the disease progression, both as a direct pathological factor and as driving force for other CVD pathomechanisms, e.g., inflammation, DNA damage, vascular wall remodeling, hemodynamic stress, releasing hemoglobin from red blood cells, or increased activity of metalloproteinases [111].

All things considered, in the PCA for all tested parameters, a beneficial effect of both flavonoids and doses is observed; however, this influence was not strong enough to match the initial state, comparable to the NDM group. Additionally, despite this main effect, a curious observation was made, as the DIO50, DIO100, and HES100 groups separated from both the NDM and T1DM groups across axis PC2, which was connected to oxidative stress markers, indicating overall dose-dependent improvement across all experimental groups. Such observations would not have been possible without the multivariate approach; therefore, analyses such as PCA are valuable tools, allowing for a holistic observation of the possible outcome. A holistic, integrated approach toward analysis of parameters measured in the onset of diabetes, insulin resistance, and metabolic diseases in general (both in animals and patients) serves as an auxiliary tool used for general pattern tracking in complex analyses, as individual assays, when observed in isolation, often present an image that seems contradictory at a first glance. Hence, integration of the accumulated data in a multivariate way helps capture the general idea of the changes taking place and identify the main drivers responsible for these changes and was used in numerous studies on diabetic subjects [112,113,114,115,116,117,118].

**Figure 10:**
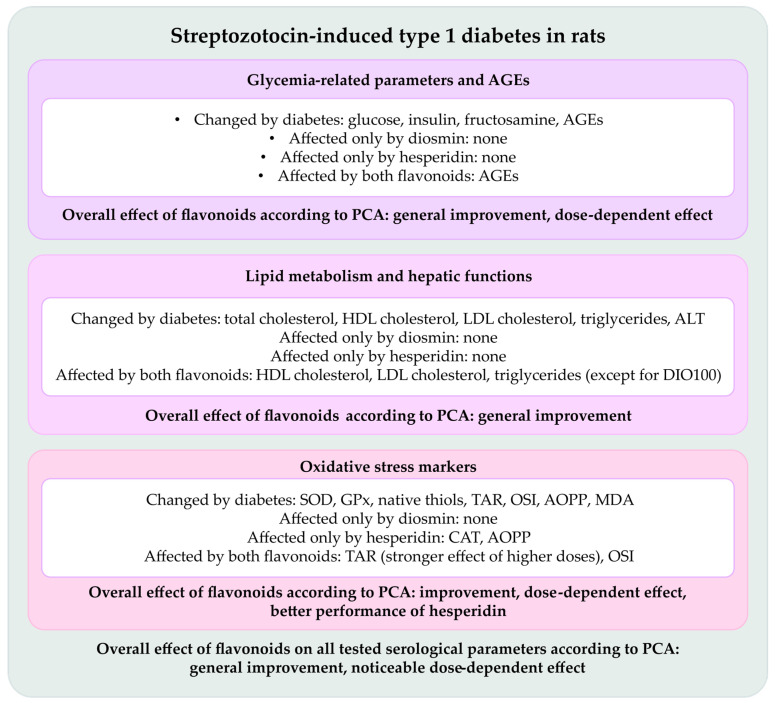
An overview on the changes caused by streptozotocin-induced type 1 diabetes in rats and effects of diosmin and hesperidin on the tested parameters. Each category of parameters is grouped in separate small boxes showing individual effects (white part of the small boxes) and aggregated effect revealed by PCA (colored part of the small boxes). Additionally, a large box encompassing all parameters sums up the overall effect of the tested substances observed based on PCA. AGEs—advanced glycation end products, ALT—alanine transaminase, AOPP—advanced oxidation protein products, CAT—catalase, DIO100—type 1 diabetic rats treated with diosmin at a dose of 100 mg/kg, HDL cholesterol—high-density lipoprotein cholesterol; LDL cholesterol—low-density lipoprotein cholesterol, MDA—malondialdehyde, OSI—oxidative stress index, PCA—principal component analysis, TAR—total antioxidative response.

Our study encompasses many serological parameters analyzed first separately, then collectively with regard to their metabolic category, and finally holistically to illuminate the whole picture of diosmin and hesperidin action. Figure 10 summarizes the results obtained in this experiment.

## 4. Materials and Methods

### 4.1. Chemicals and Kits

Streptozotocin (STZ), Superoxide dismutase kit (SOD) Item No. 706002, Catalase kit (CAT) Item No. 707002, Glutathione peroxidase kit (GPx) Item No. 703102, H_2_O_2_—Cayman Chemicals Company, Ann Arbor, MI, USA; Diosmin (DIO), Hesperidin (HES), Chloramine T, 1,1,3,3-tetraethoxypropane, Trolox, *o*-dianisidine, Xylenol orange, Thiobarbituric acid, Sodium borohydride (NaBH_4_), Methanol, Formaldehyde, Ethylenediaminetetraacetic acid (EDTA), Tris(hydroxymethyl)aminomethane (TRIS), Reduced glutathione (GSH)—Sigma Aldrich, St. Louis, MO, USA; Glucose kit, Ref. 11504, Fructosamine kit, Ref. 11046, Total cholesterol kit, Ref. 11505, HDL cholesterol kit, Ref. 11557, LDL cholesterol kit, Ref. 11585, Triglicerides kit, Ref. 11528, Aspartate transaminase kit (AST), Ref. 11531, Alanine transaminase kit (ALT), Ref. 11533, Total protein kit, Ref. 11500—BioSystems S.A., Barcelona, Spain; Acetic acid (99.9%)—POCH, Gliwice, Poland; Potassium iodide—Stanlab, Lublin, Poland; Ferrous ammonium sulfate; Trichloroacetic acid—Eurochem BGD, Tarnów, Poland; Glycerol, NaCl, H_2_SO_4_—Chempur, Piekary Śląskie, Poland; Ketamine—Ketamina 10%, Biowet Puławy, Puławy, Poland; Xylazine—Xylapan, Vetoquinol Biowet, Gorzów Wlkp., Poland; Insulin—Ultrasensitive Rat Insulin ELISA, cat. No. 10-1251-01, Mercodia AB, Uppsala, Sweden; Advanced glycation end products kit (AGEs), cat. No. STA-317—OxiSelect ELISA kit, Cell Biolabs, Inc., San Diego, CA, USA.

### 4.2. Animals and Experimental Design

The experiment was carried out on three-month-old male albino Wistar rats. The animals, provided by the Center of Experimental Medicine at the Medical University of Silesia in Katowice, were allowed to acclimatize before the main part of the experiment in standard plastic cages, with 4–5 rats per cage. The light-dark cycle was set at 12:12 h and room conditions were set in accordance with European Union guidelines (directive 2010/63/EU). Before and during the study the animals had unlimited access to drinking water and were fed with Labofeed B standard laboratory chow (Wytwórnia Pasz “Morawski”, Kcynia, Poland). All procedures were approved by the Local Ethics Committee in Katowice, Poland (approval no. 36/2015; 18 March 2015).

After the acclimatization period, the rats were assigned into following experimental groups:

- NDM—control rats in which diabetes was not induced (nondiabetic rats)
- T1DM—control rats in which type 1 diabetes was induced
- DIO50—type 1 diabetic rats administered with diosmin at a dose of 50 mg/kg
- DIO100—type 1 diabetic rats administered with diosmin at a dose of 100 mg/kg
- HES50—type 1 diabetic rats administered with hesperidin at a dose of 50 mg/kg
- HES100—type 1 diabetic rats administered with hesperidin at a dose of 100 mg/kg

Type 1 diabetes in all diabetic groups was induced by a single injection of freshly prepared streptozotocin dissolved in a 0.1 M, pH 4.5 citrate buffer at a dose of 60 mg/kg of body weight—the volume of injection was adjusted to every rat according to its actual weight. Two weeks after STZ treatment the blood glucose level was evaluated. The tip of the tail of each animal was punctured, and a drop of capillary blood was collected on a strip and measured in a MicroDot glucometer (Cambridge Sensor USA, Plainfield, IL, USA). Only rats with a blood glucose level exceeding 200 mg/dL (11.1 mmol/L) were subjected to further stages of the study and were administered with an appropriate drug (or water in the T1DM group). In order to maintain the same conditions of the experiment in all the experimental groups, the NDM rats were also subjected to glucose measurement protocol.

NDM and T1DM groups were administered with water by intragastric tube at a volume adjusted to the actual body weight of each rat. Diosmin and hesperidin were suspended in water in order to obtain a dose of 50 or 100 mg/kg, and adequate suspension was given to rats in a suitable group at a volume of 1 mL/kg, according to the body weight of each rat. Drugs (and water in control groups) were administered for 28 consecutive days. The doses of the flavonoids were chosen based on the literature data [60,119,120,121].

One day after the drug administration period all rats were euthanized by sedation induced by injection of a ketamine and xylazine mixture (87.5 mg/kg + 12.5 mg/kg, respectively) followed by cardiac perfusion and collection of the total blood volume from the heart. Obtained blood was subsequently centrifuged in order to collect the serum required for biochemical analyses. When not used, serum was stored in a freezer. All biochemical assays were measured in a microplate reader Tecan Infinite M200 PRO equipped with Magellan 7.2 software (Tecan Austria, Grödig, Austria).

### 4.3. Carbohydrate Metabolism Markers and Advanced Glycation End-Product Analysis

In the obtained serum, the following markers related to carbohydrate metabolism were measured: glucose level, insulin level and fructosamine level using commercially available colorimetric and ELISA kits. Advanced glycation end products (AGEs) level was assayed using an ELISA kit.

### 4.4. Lipid Metabolism Hepatic Function Markers Analysis

Total cholesterol, LDL cholesterol, HDL cholesterol, and triglyceride levels were measured using commercially available colorimetric kits. Moreover, AST and ALT activity was measured using colorimetric kits. All reactions were performed according to the manufacturers’ manuals.

### 4.5. Antioxidative Enzymes Activity Analysis

The activity of superoxide dismutase (SOD), catalase (CAT), and glutathione peroxidase (GPx) was evaluated in the serum with adequate colorimetric kits according to user manuals. The results for enzymes activity were standardized per total protein content in the serum which was measured with a commercially available colorimetric kit, according to the attached manual.

### 4.6. Oxidative Damage Markers Analysis

Serological advanced oxidation protein products (AOPP) were evaluated according to the method presented by Witko-Sarsat et al. with potassium iodide and anhydrous acetic acid as reagents and chloramine T as a standard, measured at 340 nm [122]. The malondialdehyde (MDA) level in the serum was estimated according to the method presented by Ohkawa et al. exploiting thiobarbituric acid in acidic environment as a reactive substance responsible for color development and with 1,1,3,3-tetraethoxypropane used as a reference [123]. The reaction was read at 532 nm.

### 4.7. Oxidative Stress Status Analysis

Total antioxidative response (TAR) and total oxidative status (TOS) in the serum were measured according to methods presented by Erel. In the TAR method, all samples were mixed with *o*-dianisidine and ferrous ammonium sulfate diluted in Clark and Lubs solution. The mixture was read at 444 nm; then, another measurement at 444 nm after 4 min incubation with 7.5 mM H_2_O_2_ solution in Clark and Lubs solution was taken. Trolox was used as a standard [124]. TOS method was based on mixing the samples with xylenol orange, NaCl, and sulfuric acid and reading the reaction at main wavelength 560 nm and reference wavelength 800 nm. Subsequently, ferrous ammonium sulfate with *o*-dianisidine was added, and after four minutes the read was taken at the same wavelengths. H_2_O_2_ was used as a standard [125]. Based on TAR and TOS results, the oxidative stress index (OSI) was calculated as follows: OSI = TOS/(TAR × 100) [126].

### 4.8. Thiols Content Analysis

An automated method for thiol/disulfide homeostasis developed by Erel and Neselioglu was applied for measurement of total thiols, native thiols, and disulfides. Total thiols were measured by adding to the samples 10 mM NaBH_4_ solution dissolved in a methanol:water 50:50 mixture; then a mixture consisting of 6.715 mM formaldehyde and 10 mM EDTA in 100 mM TRIS buffer (pH 8.2) was added. Next, the read at 415 nm (with 700 nm as reference) wavelength was made. Subsequently, 10 mM DTNB in methanol was added to the samples and the measurement at 415 nm and 700 nm wavelengths was repeated. Samples treatment for native thiols differed from the total thiols measurement by adding 10 mM NaCl solution dissolved in methanol:water 50:50 *v*/*v* mixture instead of NaBH_4_. All further steps were identical. The standard curve was prepared with GSH. Disulfides were calculated as half of the difference between total and native thiols [127].

### 4.9. Statistical Analysis

The data is presented as arithmetic mean ± standard deviation (SD); NDM: n = 9, T1DM: n = 8, DIO50: n = 9, DIO100: n = 9, HES50: n = 8, HES100: n = 9. The results were evaluated in the Statistica 13.3 (TIBCO Software Inc.) software with one-way analysis of variance (ANOVA) followed by Fisher’s Least Significant Difference (LSD) post hoc test.

The obtained data was also subjected to PCA (principal component analysis), which is a multivariate statistical analysis. Large datasets of different variables were transformed into a two-dimensional plot presented on a plane defined by principal component axes to identify the underlying patterns of variation between the obtained samples and parameters. Covariance matrix of the dataset was used to identify the major sources of variation responsible for the distribution of individual data points scattered between the PC1 and PC2 axes. For better data granularity and clarity of the presentation, four PCA plots were prepared, including all parameters obtained from the serum, as well as markers of the glycemic state, oxidative stress, and lipid-associated variables. The significance of observed results was tested with MANOVA. PCA was calculated in the Past 5 software [128], while MANOVA in the Statistica software.

## 5. Conclusions

Diosmin and hesperidin, two venoactive drugs commonly used in chronic venous disease, reveal beneficial effects in type 1 diabetic rats. This outcome is visible with regards to oxidative stress, where dose-dependency of the tested flavonoids and better potency of hesperidin was noted. A holistic analysis of various aspects related to diabetes, which are also important from a CVD pathogenesis point of view, suggests that both diosmin and hesperidin could be important adjunct agents averting negative impact of diabetes via their multifactorial activity.

Since our research bears several limitations, such as a lack of molecular analyses of the signaling pathways orchestrating the antioxidative responses of diosmin and hesperidin or their effects on crucial organs connected with carbohydrate metabolism (e.g., pancreas) or lipid metabolism and antioxidative defense of the body (e.g., liver), the findings revealed in this study should be further investigated in order to understand the potential mechanisms underlying the observed effects.

## Figures and Tables

**Table 1:** Principal component analysis of carbohydrate metabolism parameters and AGEs level measured in the serum of type 1 diabetic rats after diosmin and hesperidin treatment.

	NDM	T1DM	DIO50	DIO100	HES50	HES100
PC1	−2.78 ± 0.88 ^d^	1.24 ± 0.54 ^a^	0.75 ± 0.51 ^ab^	−0.19 ± 0.85 ^c^	0.78 ± 0.55 ^ab^	0.31 ± 0.60 ^bc^
PC2	0.32 ± 1.23 ^ab^	0.90 ± 0.78 ^a^	−0.34 ± 0.67 ^b^	−0.17 ± 0.83 ^b^	−0.28 ± 0.46 ^b^	−0.36 ± 0.90 ^b^

NDM—control rats in which diabetes was not induced (n = 9), T1DM—control rats in which type 1 diabetes was induced (n = 8), DIO50—type 1 diabetic rats administered with diosmin at a dose of 50 mg/kg (n = 9), DIO100—type 1 diabetic rats administered with diosmin at a dose of 100 mg/kg (n = 9), HES50—type 1 diabetic rats administered with hesperidin at a dose of 50 mg/kg (n = 8), HES100—type 1 diabetic rats administered with hesperidin at a dose of 100 mg/kg (n = 9), PC 1—principal component 1, and PC 2—principal component 2. Results are presented as arithmetical means ± standard deviation. Statistical significances were evaluated with MANOVA followed by Fisher’s LSD post hoc test. The letters in the superscripts indicate statistical significances. Values presented in individual rows sharing at least one letter reveal no statistically significant differences at *p* < 0.05.

**Table 2:** Principal component analysis of lipid metabolism and hepatic functions parameters measured in the serum of type 1 diabetic rats after diosmin and hesperidin treatment.

	NDM	T1DM	DIO50	DIO100	HES50	HES100
PC1	−2.03 ± 0.56 ^c^	1.59 ± 0.62 ^a^	−0.13 ± 0.91 ^b^	0.17 ± 1.04 ^b^	−0.03 ± 1.10 ^b^	0.50 ± 1.25 ^b^
PC2	−0.15 ± 0.34 ^abc^	−1.02 ± 1.08 ^c^	0.26 ± 0.66 ^ab^	−0.26 ± 0.76 ^bc^	−0.02 ± 1.26 ^ab^	0.71 ± 1.35 ^a^

NDM—control rats in which diabetes was not induced (n = 9), T1DM—control rats in which type 1 diabetes was induced (n = 8), DIO50—type 1 diabetic rats administered with diosmin at a dose of 50 mg/kg (n = 9), DIO100—type 1 diabetic rats administered with diosmin at a dose of 100 mg/kg (n = 9), HES50—type 1 diabetic rats administered with hesperidin at a dose of 50 mg/kg (n = 8), HES100—type 1 diabetic rats administered with hesperidin at a dose of 100 mg/kg (n = 9), PC 1—principal component 1, and PC 2—principal component 2. Results are presented as arithmetical means ± standard deviation. Statistical significances were evaluated with MANOVA followed by Fisher’s LSD post hoc test. The letters in the superscripts indicate statistical significances. Values presented in individual rows sharing at least one letter reveal no statistically significant differences at *p* < 0.05.

**Table 3:** Effect of diosmin and hesperidin on antioxidative enzyme activity and thiol content in the serum of type 1 diabetic rats.

	NDM	T1DM	DIO50	DIO100	HES50	HES100
SOD [U/mg of protein]	0.24 ± 0.03 ^a^	0.19 ± 0.02 ^b^	0.19 ± 0.03 ^b^	0.20 ± 0.03 ^b^	0.20 ± 0.04 ^b^	0.20 ± 0.04 ^b^
CAT [nmol/min/mg of protein]	0.48 ± 0.13 ^cb^	0.30 ± 0.11 ^c^	0.32 ± 0.12 ^c^	0.42 ± 0.15 ^c^	0.90 ± 0.59 ^a^	0.81 ± 0.60 ^ab^
GPx [nmol/min/mg of protein]	39.68 ± 9.19 ^a^	20.18 ± 3.08 ^b^	21.42 ± 3.33 ^b^	23.40 ± 4.05 ^b^	25.98 ± 5.37 ^b^	26.29 ± 5.79 ^b^
Total thiols [nmol/L]	99.50 ± 23.13	117.18 ± 17.97	113.78 ± 19.70	114.84 ± 25.84	107.75 ± 27.30	118.47 ± 19.25
Native thiols [nmol/L]	37.17 ± 14.90 ^b^	63.46 ± 20.86 ^a^	61.75 ± 19.42 ^a^	66.56 ± 33.25 ^a^	54.18 ± 27.33 ^ab^	76.50 ± 18.61 ^a^
Disulfides [nmol/L]	31.17 ± 9.63 ^a^	26.86 ± 5.73 ^ab^	26.01 ± 3.24 ^ab^	24.14 ± 5.53 ^b^	26.79 ± 8.95 ^ab^	20.99 ± 2.49 ^b^
Native thiols/ disulfides ratio	1.28 ± 0.57 ^c^	2.51 ± 1.02 ^abc^	2.41 ± 0.85 ^bc^	3.19 ± 2.32 ^ab^	2.19 ± 1.18 ^bc^	3.68 ± 1.01 ^a^

NDM—control rats in which diabetes was not induced (n = 9), T1DM—control rats in which type 1 diabetes was induced (n = 8), DIO50—type 1 diabetic rats administered with diosmin at a dose of 50 mg/kg (n = 9), DIO100—type 1 diabetic rats administered with diosmin at a dose of 100 mg/kg (n = 9), HES50—type 1 diabetic rats administered with hesperidin at a dose of 50 mg/kg (n = 8), HES100—type 1 diabetic rats administered with hesperidin at a dose of 100 mg/kg, SOD—superoxide dismutase, CAT—catalase, GPx—glutathione peroxidase. Results are presented as arithmetical means ± standard deviation. Statistical significances were evaluated with one-way ANOVA followed by Fisher’s LSD post hoc test. The letters in the superscripts indicate statistical significances. Values presented in individual rows sharing at least one letter reveal no statistically significant differences at *p* < 0.05.

**Table 4:** Principal component analysis of oxidative stress-related parameters measured in the serum of type 1 diabetic rats after diosmin and hesperidin treatment.

	NDM	T1DM	DIO50	DIO100	HES50	HES100
PC1	−2.76 ± 1.07 ^c^	1.12 ± 1.34 ^a^	0.78 ± 1.36 ^ab^	0.85 ± 1.83 ^ab^	−0.48 ± 1.63 ^b^	0.87 ± 1.22 ^ab^
PC2	−0.11 ± 0.95 ^bc^	1.69 ± 1.03 ^a^	0.75 ± 1.43 ^ab^	−0.76 ± 1.58 ^cd^	0.29 ± 1.51 ^bc^	−1.52 ± 1.35 ^d^

NDM—control rats in which diabetes was not induced (n = 9), T1DM—control rats in which type 1 diabetes was induced (n = 8), DIO50—type 1 diabetic rats administered with diosmin at a dose of 50 mg/kg (n = 9), DIO100—type 1 diabetic rats administered with diosmin at a dose of 100 mg/kg (n = 9), HES50—type 1 diabetic rats administered with hesperidin at a dose of 50 mg/kg (n = 8), HES100—type 1 diabetic rats administered with hesperidin at a dose of 100 mg/kg (n = 9), PC 1—principal component 1, and PC 2—principal component 2. Results are presented as arithmetical means ± standard deviation. Statistical significances were evaluated with MANOVA followed by Fisher’s LSD post hoc test. The letters in the superscripts indicate statistical significances. Values presented in individual rows sharing at least one letter reveal no statistically significant differences at *p* < 0.05.

**Table 5:** Principal component analysis of all tested parameters measured in the serum of type 1 diabetic rats after diosmin and hesperidin treatment.

	NDM	T1DM	DIO50	DIO100	HES50	HES100
PC1	−4.43 ± 0.83 ^c^	2.54 ± 1.23 ^a^	0.96 ± 1.21 ^b^	0.40 ± 1.64 ^b^	0.24 ± 1.44 ^b^	0.71 ± 0.83 ^b^
PC2	−0.72 ± 1.02 ^cd^	−1.83 ± 0.92 ^d^	−0.23 ± 1.34 ^bc^	1.00 ± 1.81 ^ab^	−0.43 ± 1.67 ^cd^	1.92 ± 1.53 ^a^

NDM—control rats in which diabetes was not induced (n = 9), T1DM—control rats in which type 1 diabetes was induced (n = 8), DIO50—type 1 diabetic rats administered with diosmin at a dose of 50 mg/kg (n = 9), DIO100—type 1 diabetic rats administered with diosmin at a dose of 100 mg/kg (n = 9), HES50—type 1 diabetic rats administered with hesperidin at a dose of 50 mg/kg (n = 8), HES100—type 1 diabetic rats administered with hesperidin at a dose of 100 mg/kg (n = 9), PC 1—principal component 1, and PC 2—principal component 2. Results are presented as arithmetical means ± standard deviation. Statistical significances were evaluated with MANOVA followed by Fisher’s LSD post hoc test. The letters in the superscripts indicate statistical significances. Values presented in individual rows sharing at least one letter reveal no statistically significant differences at *p* < 0.05.

## Data Availability

Data available on request.

## Abbreviations

The following abbreviations are used in this manuscript:

AGEs	Advanced glycation end products
ALT	Alanine transaminase
AOPP	Advanced oxidation protein products
AST	Asparagine transaminase
CAT	Catalase
CVD	Chronic venous disease
CVI	Chronic venous insufficiency
DIO50	Type 1 diabetic rats treated with diosmin at a dose of 50 mg/kg
DIO100	Type 1 diabetic rats treated with diosmin at a dose of 100 mg/kg
DM	Diabetes mellitus
GPx	Glutathione peroxidase
GSH	Glutathione
HDL	High-density lipoprotein
HES50	Type 1 diabetic rats treated with hesperidin at a dose of 50 mg/kg
HES100	Type 1 diabetic rats treated with hesperidin at a dose of 100 mg/kg
LDL	Low-density lipoprotein
MDA	Malondialdehyde
NDM	Nondiabetic control rats
OSI	Oxidative stress index
PC1	Principal component 1
PC2	Principal component 2
PCA	Principal component analysis
ROS	Reactive oxygen species
SOD	Superoxide dismutase
T1DM	Type 1 diabetic control rats
TAR	Total antioxidative response
TOS	Total oxidative status

## References

[1] Domingueti C.P., Dusse L.M.S., Carvalho M.D.G., De Sousa L.P., Gomes K.B., Fernandes A.P. (2016). Diabetes mellitus: The linkage between oxidative stress, inflammation, hypercoagulability and vascular complications. J. Diabetes Complicat..

[2] Guo H., Wu H., Li Z. (2023). The pathogenesis of diabetes. Int. J. Mol. Sci..

[3] Gupta S., Sharma N., Arora S., Verma S. (2024). Diabetes: A review of its pathophysiology, and advanced methods of mitigation. Curr. Med. Res. Opin..

[4] Templer S., Abdo S., Wong T. (2024). Preventing diabetes complications. Intern. Med. J..

[5] Gastaldi G., Pannier F., Roztočil K., Lugli M., Mansilha A., Haller H., Rabe E., Van Rijn M.J. (2021). Chronic venous disease and diabetic microangiopathy: Pathophysiology and commonalities. Int. Angiol..

[6] Ortega M.A., Fraile-Martínez O., García-Montero C., Álvarez-Mon M.A., Chaowen C., Ruiz-Grande F., Pekarek L., Monserrat J., Asúnsolo A., García-Honduvilla N. (2021). Understanding chronic venous disease: A critical overview of its pathophysiology and medical management. J. Clin. Med..

[7] Castro-Ferreira R., Cardoso R., Leite-Moreira A., Mansilha A. (2018). The role of endothelial dysfunction and inflammation in chronic venous disease. Ann. Vasc. Surg..

[8] González P., Lozano P., Ros G., Solano F. (2023). Hyperglycemia and oxidative stress: An integral, updated and critical overview of their metabolic interconnections. Int. J. Mol. Sci..

[9] Black H.S. (2022). A synopsis of the associations of oxidative stress, ROS, and antioxidants with diabetes mellitus. Antioxidants.

[10] Jarošíková R., Roztočil K., Husáková J., Dubský M., Bém R., Wosková V., Fejfarová V. (2023). Chronic venous disease and its intersections with diabetes mellitus. Physiol. Res..

[11] Okley D.V. (2015). Systemic phlebotropic drugs in pharmacotherapy of chronic venous insufficiency of the lower extremities. News Farm..

[12] Chaitidis N., Kokkinidis D.G., Papadopoulou Z., Kyriazopoulou M., Schizas D., Bakoyiannis C. (2022). Treatment of chronic venous disorder: A comprehensive review. Dermatol. Ther..

[13] Mostafa R.E., Ali D.E., El-Shiekh R.A., El-Alfy A.N., Abd El Hafeez M.S., Reda A.M., Fayek N.M. (2025). Therapeutic applications of natural products in the management of venous diseases: A comprehensive review. Inflammopharmacology.

[14] Martinez-Zapata M.J., Vernooij R.W., Simancas-Racines D., Uriona Tuma S.M., Stein A.T., Moreno Carriles R.M.M., Vargas E., Bonfill Cosp X. (2020). Phlebotonics for venous insufficiency. Cochrane Database Syst. Rev..

[15] Gama C.R.B., Nunes C.P., Steinbruch M., Suchmacher M., Gama G.F., Kaufman R., Mezitis S., Sitnoveter A.L., Ezri T.G.B., Daher J.P.L. (2025). Comparative efficacy and safety of a horse chestnut formulation vs. diosmin-hesperidin in chronic venous insufficiency: Randomized double-blind trial. Int. J. Clin. Med..

[16] Roy J., Azamthulla M., Mukkerjee D. (2020). Hesperidin and diosmin—A novel drugs. Int. J. Pharm. Res. Technol..

[17] Szymański M., Młynarek D., Szymański A., Matławska I. (2016). Simultaneous determination of diosmin and hesperidin in pharmaceuticals by RPLC using ionic liquids as mobile phase modifiers. Iran. J. Pharm. Res..

[18] Borko Y., Kovalevska I. (2019). Studies of physico-chemical and pharmaco-technological parameters of bioflavonoids diosmin and hesperidin. Sci. J. ScienceRise Pharm. Sci..

[19] Hassan M.A., Elmageed G.M.A., El-Qazaz I.G., El-Sayed D.S., El-Samad L.M., Abdou H.M. (2023). The synergistic influence of polyflavonoids from Citrus aurantifolia on diabetes treatment and their modulation of the PI3K/AKT/FOXO1 signaling pathways: Molecular docking analyses and in vivo investigations. Pharmaceutics.

[20] Cova D., De Angelis L., Giavarini F., Palladini G., Perego R. (1992). Pharmacokinetics and metabolism of oral diosmin in healthy volunteers. Int. J. Clin. Parmacol. Toxicol..

[21] Kanaze F.I., Bounartzi M.I., Georgarakis M., Niopas I. (2007). Pharmacokinetics of the citrus flavanone aglycones hesperetin and naringenin after single oral administration in human subjects. Eur. J. Clin. Nutr..

[22] Rice-Evans C.A., Miller N.J., Paganga G. (1996). Structure-antioxidant activity relationships of flavonoids and phenolic acids. Free Radic. Biol. Med..

[23] Mustafa S., Akbar M., Khan M.A., Sunita K., Parveen S., Pawar J.S., Massey S., Agarwal N.R., Husain S.A. (2022). Plant metabolite diosmin as the therapeutic agent in human diseases. Curr. Res. Pharmacol. Drug Discov..

[24] Huwait E., Mobashir M. (2022). Potential and therapeutic roles of diosmin in human diseases. Biomedicines.

[25] Mirzaei A., Mirzaei A., Khalilabad S.N., Askari V.R., Rahimi V.B. (2023). Promising influences of hesperidin and hesperetin against diabetes and its complications: A systematic review of molecular, cellular, and metabolic effects. EXCLI J..

[26] Rajasekar M., Baskaran P., Mary J., Sivakumar M., Selvam M. (2024). Revisiting diosmin for their potential biological properties and applications. Carbohydr. Polym. Technol. Appl..

[27] Li C., Schluesener H. (2017). Health-promoting effects of the citrus flavanone hesperidin. Crit. Rev. Food Sci. Nutr..

[28] Vinayagam R., Xu B. (2015). Antidiabetic properties of dietary flavonoids: A cellular mechanism review. Nutr. Metab..

[29] Gerges S.H., Wahdan S.A., Elsherbiny D.A., El-Demerdash E. (2022). Pharmacology of diosmin, a citrus flavone glycoside: An updated review. Eur. J. Drug Metab. Pharmacokinet..

[30] Wdowiak K., Walkowiak J., Pietrzak R., Bazan-Woźniak A., Cielecka-Piontek J. (2022). Bioavailability of hesperidin and its aglycone hesperetin—Compounds found in citrus fruits as a parameter conditioning the pro-health potential (neuroprotective and antidiabetic activity)—Mini-review. Nutrients.

[31] Otto-Buczkowska E., Jainta N. (2018). Pharmacological treatment in diabetes mellitus type 1—Insulin and what else ?. Int. J. Endocrinol. Metab..

[32] Lefever E., Vliebergh J., Mathieu C. (2021). Improving the treatment of patients with diabetes using insulin analogues: Current findings and future directions. Expert Opin. Drug Saf..

[33] Dahiru M.M., Nadro M.S. (2022). A review of the mechanisms of action and side effects of anti-diabetic agents. Trends Pharmacol. Sci..

[34] Dilworth L., Stennett D., Facey A., Omoruyi F., Mohansingh S., Omoruyi F.O. (2024). Diabetes and the associated complications: The role of antioxidants in diabetes therapy and care. Biomed. Pharmacother..

[35] Wang W., Wang X., Cao S., Duan Y., Xu C., Gan D., He W. (2022). Dietary antioxidant indices in relation to all-cause and cause-specific mortality among adults with diabetes: A prospective cohort study. Front. Nutr..

[36] Hsu C.C., Lin M.H., Cheng J.T., Wu M.C. (2017). Diosmin, a citrus nutrient, activates imidazoline receptors to alleviate blood glucose and lipids in type 1-like diabetic rats. Nutrients.

[37] Hsu C.C., Lin M.H., Cheng J.T., Wu M.C. (2017). Antihyperglycaemic action of diosmin, a citrus flavonoid, is induced through endogenous β-endorphin in type I-like diabetic rats. Clin. Exp. Pharmacol. Physiol..

[38] Akiyama S., Katsumata S., Suzuki K., Ishimi Y., Wu J., Uehara M. (2010). Hypoglycemic and hypolipidemic effects dietary hesperidin exerts in streptozotocin-induced marginal type 1 diabetic rats. J. Clin. Biochem. Nutr..

[39] Shehata A.S., Mohamed D.A., Hagras S.M., El-Beah S.M., Elnegris H.M. (2021). The role of hesperidin in ameliorating retinal changes in rats with experimentally induced type 1 diabetes mellitus and the active role of vascular endothelial growth factor and glial fibrillary acidic protein. Anat. Cell Biol..

[40] Shehata A.S., Amer M.G., Abd El-Haleem M.R., Karam R.A. (2017). The ability of hesperidin compared to that of insulin for preventing osteoporosis induced by type I diabetes in young male albino rats: A histological and biochemical study. Exp. Toxicol. Pathol..

[41] Kandemir F.M., Ozkaraca M., Küçükler S., Caglayan C., Hanedan B. (2018). Preventive effects of hesperidin on diabetic nephropathy induced by streptozotocin via modulating TGF-β1 and oxidative DNA damage. Toxin Rev..

[42] Keenoy B.M., Vetrmmen J., de Leeuw I. (1999). The effect of flavonoid treatment on the glycation and antioxidant status in type 1 diabetic patients. Diabetes Nutr. Metab..

[43] Carballo-Villalobos A.I., González-Trujano M.-E., Pellicer F., López-Muñoz F.J. (2016). Antihyperalgesic effect of hesperidin improves with diosmin in experimental neuropathic pain. BioMed Res. Int..

[44] Yang M., Tanaka T., Hirose Y., Deguchi T., Mori H., Kawada Y. (1997). Chemopreventive effects of diosmin and hesperidin on n-butyl-n-(4-hydroxybutyl) nitrosamine-induced urinary-bladder carcinogenesis in male ICR mice. Int. J. Cancer.

[45] Tanaka T., Makita H., Kawabata K., Mori H., Kakumoto M., Satoh K., Hara A., Sumida T., Tanaka T., Ogawa H. (1997). Chemoprevention of azoxymethane-induced rat colon carcinogenesis by the naturally occurring flavonoids, diosmin and hesperidin. Carcinogenesis.

[46] Tanaka T., Makita H., Kawabata K., Mori H., Kakumoto M., Satoh K., Hara A., Sumida T., Fukutani K., Tanaka T. (1997). Modulation of N-methyl-N-amylnitrosamine-induced rat oesophageal tumourigenesis by dietary feeding of diosmin and hesperidin, both alone and in combination. Carcinogenesis.

[47] Tanaka T., Makita H., Ohnishi M., Mori H., Satoh K., Hara A., Sumida T., Fukutani K., Tanaka T., Ogawa H. (1997). Chemoprevention of 4-nitroquinoline 1-oxide-induced oral carcinogenesis in rats by flavonoids diosmin and hesperidin, each alone and in combination. Cancer Res..

[48] Elhelaly A.E., AlBasher G., Alfarraj S., Almeer R., Bahbah E.I., Fouda M.M.A., Bungău S.G., Aleya L., Abdel-Daim M.M. (2019). Protective effects of hesperidin and diosmin against acrylamide-induced liver, kidney, and brain oxidative damage in rats. Environ. Sci. Pollut. Res..

[49] Bednarska K., Fecka I. (2021). Potential of vasoprotectives to inhibit non-enzymatic protein glycation, and reactive carbonyl and oxygen species uptake. Int. J. Mol. Sci..

[50] Pinna C., Sala A. (2019). Sex-specific activity of hesperidin, diosmin and genistein on human umbilical vein. Biomed. Res. Clin. Pract. Res..

[51] Osama H., Hamed E.O., Mahmoud M.A., Abdelrahim M.E.A. (2023). The effect of hesperidin and diosmin individually or in combination on metabolic profile and neuropathy among diabetic patients with metabolic syndrome: A randomized controlled trial. J. Diet. Suppl..

[52] Borymska W., Borymski S., Zych M., Kaczmarczyk-Żebrowska I. Porównanie potencjału antyoksydacyjnego dwóch flawonoidów flebotropowych—Diosminy i hesperydyny—W przebiegu cukrzycy typu 1. Proceedings of the 7 Śląskie Farmaceutyczne Spotkanie Naukowe “Od Nauki do Pacjenta”.

[53] Issaq H.J., Xiao Z., Veenstra T.D. (2007). Serum and plasma proteomics. Chem. Rev..

[54] Liu L., Aa J., Wang G., Yan B., Zhang Y., Wang X., Zhao C., Cao B., Shi J., Li M. (2010). Differences in metabolite profile between blood plasma and serum. Anal. Biochem..

[55] Szkudelski T. (2001). The mechanism of alloxan and streptozotocin action in B cells of the rat pancreas. Physiol. Res..

[56] Lenzen S. (2008). The mechanisms of alloxan- and streptozotocin-induced diabetes. Diabetologia.

[57] Klasik-Ciszewska S., Londzin P., Grzywnowicz K., Borymska W., Zych M., Kaczmarczyk-Żebrowska I., Folwarczna J. (2025). Effect of chrysin, a flavonoid present in food, on the skeletal system in rats with experimental type 1 diabetes. Nutrients.

[58] Wojnar W., Kaczmarczyk-Sedlak I., Zych M. (2017). Diosmin ameliorates the effects of oxidative stress in lenses of streptozotocin-induced type 1 diabetic rats. Pharmacol. Rep..

[59] Sundaram R., Nandhakumar E., Haseena Banu H. (2019). Hesperidin, a citrus flavonoid ameliorates hyperglycemia by regulating key enzymes of carbohydrate metabolism in streptozotocin-induced diabetic rats. Toxicol. Mech. Methods.

[60] Pari L., Srinivasan S. (2010). Antihyperglycemic effect of diosmin on hepatic key enzymes of carbohydrate metabolism in streptozotocin-nicotinamide-induced diabetic rats. Biomed. Pharmacother..

[61] Malmström H., Walldius G., Grill V., Jungner I., Gudbjörnsdottir S., Hammar N. (2014). Fructosamine is a useful indicator of hyperglycaemia and glucose control in clinical and epidemiological studies—Cross-sectional and longitudinal experience from the AMORIS cohort. PLoS ONE.

[62] Shams-Rad S., Mohammadi M., Ramezani-Jolfaie N., Zarei S., Mohsenpour M., Salehi-Abargouei A. (2020). Hesperidin supplementation has no effect on blood glucose control: A systematic review and meta-analysis of randomized controlled clinical trials. Br. J. Clin. Pharmacol..

[63] Takeuchi M., Sakasai-Sakai A., Takata T., Takino J., Koriyama Y. (2022). Effects of toxic AGEs (TAGE) on human health. Cells.

[64] Kuzan A. (2021). Toxicity of advanced glycation end products (Review). Biomed. Rep..

[65] Khalid M., Petroianu G., Adem A. (2022). Advanced glycation end products and diabetes mellitus: Mechanisms and perspectives. Biomolecules.

[66] Hafizur R.M., Momin S., Fatima N. (2017). Prevention of advanced glycation end-products formation in diabetic rats through beta-cell modulation by Aegle marmelos. BMC Complement. Altern. Med..

[67] Li L., Song Q., Zhang X., Yan Y., Wang X. (2022). Allicin alleviates diabetes mellitus by inhibiting the formation of advanced glycation end products. Molecules.

[68] El-Fawal R., El Fayoumi H.M., Mahmoud M.F. (2019). Effects of diosmin and crocin on metabolic syndrome-associated cardio-vascular complications in rats. Naunyn. Schmiedebergs. Arch. Pharmacol..

[69] Patil K.K., Meshram R.J., Dhole N.A., Gacche R.N. (2016). Role of dietary flavonoids in amelioration of sugar induced cataractogenesis. Arch. Biochem. Biophys..

[70] Khan M.S., Rehman M.T., Ismael M.A., AlAjmi M.F., Alruwaished G.I., Alokail M.S., Khan M.R. (2021). Bioflavonoid (hesperidin) restrains protein oxidation and advanced glycation end product formation by targeting AGEs and glycolytic enzymes. Cell Biochem. Biophys..

[71] Wang Y., Wang L., Xu G., Wei D. (2019). Hesperidin exerts the gestational diabetes mellitus via AGEs-RAGE signalling pathway. Int. J. Pharmacol..

[72] Shi X., Liao S., Mi H., Guo C., Qi D., Li F., Zhang C., Yang Z. (2012). Hesperidin prevents retinal and plasma abnormalities in streptozotocin-induced diabetic rats. Molecules.

[73] Lee J., Yun J.S., Ko S.H. (2022). Advanced glycation end products and their effect on vascular complications in type 2 diabetes mellitus. Nutrients.

[74] Rhee S.Y., Kim Y.S. (2018). The role of advanced glycation end products in diabetic vascular complications. Diabetes Metab. J..

[75] Bierhansl L., Conradi L.-C., Treps L., Dewerchin M., Carmeliet P. (2017). Central role of metabolism in endothelial cell function and vascular disease. Physiology.

[76] Yamagishi S., Nakamura N., Suematsu M., Kaseda K., Matsui T. (2015). Advanced glycation end products: A molecular target for vascular complications in diabetes. Mol. Med..

[77] Vergès B. (2020). Dyslipidemia in type 1 diabetes: A masked danger. Trends Endocrinol. Metab..

[78] Athyros V.G., Doumas M., Imprialos K.P., Stavropoulos K., Georgianou E., Katsimardou A., Karagiannis A. (2018). Diabetes and lipid metabolism. Hormones.

[79] Haffar S., Izzy M., Habib H., Sugihara T., Li D.K., Sharma A., Wang Z., Murad M.H., Watt K.D., Bazerbachi F. (2021). Liver chemistries in glycogenic hepatopathy associated with type 1 diabetes mellitus: A systematic review and pooled analysis. Liver Int..

[80] Ozkol H., Tuluce Y., Dilsiz N., Koyuncu I. (2013). Therapeutic potential of some plant extracts used in turkish traditional medicine on streptozocin-induced type 1 diabetes mellitus in rats. J. Membr. Biol..

[81] Komeili G., Hashemi M., Bameri-Niafar M. (2016). Evaluation of antidiabetic and antihyperlipidemic effects of peganum harmala seeds in diabetic rats. Cholesterol.

[82] Ahmadvand H., Ghasemi-Dehnoo M. (2014). Antiatherogenic, hepatoprotective, and hypolipidemic effects of coenzyme Q10 in alloxan-induced type 1 diabetic rats. ARYA Atheroscler..

[83] Srinivasan S., Pari L. (2013). Antihyperlipidemic effect of diosmin: A citrus flavonoid on lipid metabolism in experimental diabetic rats. J. Funct. Foods.

[84] Liu C., Hao S., Zhang M., Wang X., Chu B., Wen T., Dang R., Sun H. (2025). Natural diosmin alleviating obesity and nonalcoholic fatty liver disease by regulating the activating the AMP-activated protein kinase (AMPK) pathway. Chin. J. Nat. Med..

[85] Yıldızhan K., Bayir M.H., Huyut Z., Altındağ F. (2025). Effect of hesperidin on lipid profile, inflammation and apoptosis in experimental diabetes. Dokl. Biochem. Biophys..

[86] Ahmed O.M., Mahmoud A.M., Abdel-Moneim A., Ashour M.B. (2012). Antidiabetic effects of hesperidin and naringin in type 2 diabetic rats. Diabetol. Croat..

[87] Wu C.-H., Lin J.-A., Hsieh W.-C., Yen G.-C. (2009). Low-Density-Lipoprotein (LDL)-bound flavonoids increase the resistance of LDL to oxidation and glycation under pathophysiological concentrations of glucose in vitro. J. Agric. Food Chem..

[88] Doğduş M., Koç A. (2020). Higher serum Endocan levels are involved in the pathophysiology of chronic venous insufficiency. Ege Tıp Derg..

[89] Papachristoforou E., Lambadiari V., Maratou E., Makrilakis K. (2020). Association of glycemic indices (hyperglycemia, glucose variability, and hypoglycemia) with oxidative stress and diabetic complications. J. Diabetes Res..

[90] Lushchak V.I. (2016). Free radicals, reactive oxygen species, oxidative stresses and their classifications. Chem. Biol. Interact..

[91] Ali M.M., Agha F.G. (2009). Amelioration of streptozotocin-induced diabetes mellitus, oxidative stress and dyslipidemia in rats by tomato extract lycopene. Scand. J. Clin. Lab. Investig..

[92] Kabay S.C., Ozden H., Guven G., Ustuner M.C., Degirmecni I., Olgun E.G., Unal N. (2009). Protective effects of vitamin E on central nervous system in streptozotocin-induced diabetic rats. Clin. Investig. Med..

[93] Grabia M., Socha K., Soroczyńska J., Bossowski A., Markiewicz-Żukowska R. (2023). Determinants related to oxidative stress parameters in pediatric patients with type 1 diabetes mellitus. Nutrients.

[94] Alghazeer R., Alghazir N., Awayn N., Ahtiwesh O., Elgahmasi S. (2018). Biomarkers of oxidative stress and antioxidant defense in patients with type 1 diabetes mellitus. Ibnosina J. Med. Biomed. Sci..

[95] Yildirim Z., Yildirim F., Ucgun N.I., Kilic N. (2009). The evaluation of the oxidative stress parameters in nondiabetic and diabetic senile cataract patients. Biol. Trace Elem. Res..

[96] Piwowar A., Knapik-Kordecka M., Warwas M. (2007). AOPP and its relations with selected markers of oxidative/antioxidative system in type 2 diabetes mellitus. Diabetes Res. Clin. Pract..

[97] Agarwal S., Tripathi R., Mohammed A., Rizvi S.I., Mishra N. (2020). Effects of thymol supplementation against type 2 diabetes in streptozotocin-induced rat model. Plant Arch..

[98] Astari L.F., Cahyono H.A., Widjajanto E. (2017). Correlation of interleukin-10, superoxide dismutase (SOD), and malondialdehyde (MDA) levels with HbA1c in pediatric type 1 diabetes mellitus. J. Trop. Life Sci..

[99] Chandran K., Lee S.M., Shen L., Tng E.L. (2024). Fructosamine and HbA1c: A correlational study in a southeast asian population. J. ASEAN Fed. Endocr. Soc..

[100] Hasan H.F., Abdel-Rafei M.K., Galal S.M. (2017). Diosmin attenuates radiation-induced hepatic fibrosis by boosting PPAR-γ expression and hampering miR-17-5p-activated canonical Wnt-β-catenin signaling. Biochem. Cell Biol..

[101] Barreca D., Laganà G., Bruno G., Magazù S., Bellocco E. (2013). Diosmin binding to human serum albumin and its preventive action against degradation due to oxidative injuries. Biochimie.

[102] Kumar R., Akhtar F., Rizvi S.I. (2020). Hesperidin attenuates altered redox homeostasis in an experimental hyperlipidaemic model of rat. Clin. Exp. Pharmacol. Physiol..

[103] Shakour N., Mahdinezhad M.R., Hadjzadeh M.A.R., Sahebkar A., Hadizadeh F. (2024). Serum biochemical evaluation following administration of imidazolyl thiazolidinedione in streptozotocin-induced diabetic rats. J. Mol. Histol..

[104] Van Laer K., Hamilton C.J., Messens J. (2013). Low-molecular-weight thiols in thiol-disulfide exchange. Antioxid. Redox Signal..

[105] Anwar M.M., Meki A.R.M.A. (2003). Oxidative stress in streptozotocin-induced diabetic rats: Effects of garlic oil and melatonin. Comp. Biochem. Physiol.—Part A.

[106] Al-Matubsi H., Rashan L., Aburayyan W., Al Hanbali O., Abuarqoub D., Efferth T. (2024). Antidiabetic and antioxidant properties of Boswellia sacra oleo-gum in streptozotocin-induced diabetic rats. J. Ayurveda Integr. Med..

[107] Salama A., Asaad G.F., Shaheen A. (2022). Chrysin ameliorates STZ-induced diabetes in rats: Possible impact of modulation of TLR4/NF-κβ pathway. Res. Pharm. Sci..

[108] Shiming Z., Mak K.K., Balijepalli M.K., Chakravarthi S., Pichika M.R. (2021). Swietenine potentiates the antihyperglycemic and antioxidant activity of metformin in streptozotocin induced diabetic rats. Biomed. Pharmacother..

[109] Hamamcioglu A.C., Severcan C., Bayraktaroglu T. (2022). Relationship of thiol/disulphide homeostasis with oxidative stress parameters in non-diabetic, prediabetic and type 2 diabetic Turkish women. Istanbul J. Pharm..

[110] Dickinson D.A., Forman H.J. (2002). Glutathione in defense and signaling: Lessons from a small thiol. Ann. N. Y. Acad. Sci..

[111] Rațiu S., Mariș M.I., Furdui-Lința A.V., Sima L.V., Bratu T.I., Sturza A., Muntean D.M., Crețu O.M. (2025). Oxidative stress in the pathophysiology of chronic venous disease: An overview. Antioxidants.

[112] Geravandi S., Emamgholipour S., Pakdaman M., Sari A.A., Esmaeili A. (2025). Principal components of type 2 diabetes risk: An exploratory factor analysis in an Iranian cohort. BMC Public Health.

[113] Yang Y., Wang L., Wang S., Huang R., Zheng L., Liang S., Zhang L., Xu J. (2014). An integrated metabonomic approach to studying metabolic profiles in rat models with insulin resistance induced by high fructose. Mol. Biosyst..

[114] Gao H., Jiang Q., Ji H., Ning J., Li C., Zheng H. (2019). Type 1 diabetes induces cognitive dysfunction in rats associated with alterations of the gut microbiome and metabolomes in serum and hippocampus. BBA—Mol. Basis Dis..

[115] Jayakumar V., Ahmed S.S.S.J., Ebenezar K.K. (2016). Multivariate analysis and molecular interaction of curcumin with PPARγ in high fructose diet induced insulin resistance in rats. Springerplus.

[116] Abdou H.M., Elmageed G.M.A., Hussein H.K., Yamari I., Chtita S., El-Samad L.M., Hassan M.A. (2025). Antidiabetic effects of quercetin and silk sericin in attenuating dysregulation of hepatic gluconeogenesis in diabetic rats through potential modulation of PI3K/Akt/FOXO1 signaling: In vivo and in silico studies. Xenobiotics.

[117] Xu H., Zhang Z., Shu Z., Jia Z. (2025). A plasma metabolomic analysis revealed the metabolic regulatory mechanism of the water extract of Dendrobium huoshanense in improving streptozotocin—Induced type 1 diabetes model rats. J. Nat. Med..

[118] Ma L., Liu J., Deng M., Zhou L., Zhang Q., Xiao X. (2024). Metabolomics analysis of serum and urine in type 1 diabetes patients with different time in range derived from continuous glucose monitoring. Diabetol. Metab. Syndr..

[119] Jain D., Bansal M.K., Dalvi R., Upganlawar A., Somani R. (2014). Protective effect of diosmin against diabetic neuropathy in experimental rats. J. Integr. Med..

[120] El-Marasy S.A., Abdallah H.M.I., El-Shenawy S.M., El-Khatib A.S., El-Shabrawy O.A., Kenawy S.A. (2014). Anti-depressant effect of hesperidin in diabetic rats. Can. J. Physiol. Pharmacol..

[121] Visnagri A., Kandhare A.D., Chakravarty S., Ghosh P., Bodhankar S.L. (2014). Hesperidin, a flavanoglycone attenuates experimental diabetic neuropathy via modulation of cellular and biochemical marker to improve nerve functions. Pharm. Biol..

[122] Witko-Sarsat V., Friedlander M., Capeillère-Blandin C., Nguyen-Khoa T., Nguyen A.T., Zingraff J., Jungers P., Descamps-Latscha B. (1996). Advanced oxidation protein products as a novel marker of oxidative stress in uremia. Kidney Int..

[123] Ohkawa H., Ohishi N., Yagi K. (1979). Assay for lipid peroxides in animal tissues by thiobarbituric acid reaction. Anal. Biochem..

[124] Erel O. (2004). A novel automated method to measure total antioxidant response against potent free radical reactions. Clin. Biochem..

[125] Erel O. (2005). A new automated colorimetric method for measuring total oxidant status. Clin. Biochem..

[126] Kosecik M., Erel O., Sevinc E., Selek S. (2005). Increased oxidative stress in children exposed to passive smoking. Int. J. Cardiol..

[127] Erel O., Neselioglu S. (2014). A novel and automated assay for thiol/disulphide homeostasis. Clin. Biochem..

[128] Hammer Ø., Harper D.A.T., Ryan P.D. (2001). Past: Paleontological statistics software package for education and data analysis. Palaeontol. Electron..

